# Evaluation of the stability of cucurbit[8]uril-based ternary host−guest complexation in physiological environment and the fabrication of a supramolecular theranostic nanomedicine

**DOI:** 10.1186/s12951-021-01076-z

**Published:** 2021-10-20

**Authors:** Han Wu, Zuobing Chen, Shaolong Qi, Bing Bai, Jiajun Ye, Dan Wu, Jie Shen, Fei Kang, Guocan Yu

**Affiliations:** 1 Key Laboratory of Organic Optoelectronics and Molecular Engineering, Department of Chemistry, Tsinghua University, Beijing, 100084 People’s Republic of China; 2 Department of Rehabilitation Medicine, The First Affiliated Hospital, College of Medicine, Zhejiang University, Hangzhou, 310003 People’s Republic of China; 3 Department of Nuclear Medicine, Xijing Hospital, Fourth Military Medical University, Xi’an, 400030 People’s Republic of China; 4 College of Materials Science and Engineering, Zhejiang University of Technology, Hangzhou, 310014 People’s Republic of China; 5 Department of Pharmacy, School of Medicine, Zhejiang University City College, Hangzhou, 310015 People’s Republic of China

**Keywords:** Supramolecular chemistry, Host–guest molecular recognition, Nanomedicine, Drug delivery, Chemotherapy

## Abstract

**Background:**

Supramolecular theranostics have exhibited promising potentials in disease diagnosis and therapy by taking advantages of the dynamic and reversible nature of non-covalent interactions. It is extremely important to figure out the stability of the driving forces in physiological environment for the preparation of theranostic systems.

**Methods:**

The host−guest complexation between cucurbit[8]uril (CB[8]), 4,4′-bipyridinium, and napththyl guest was fully studied using various characterizations, including nuclear magnetic resonance spectroscopy, ultraviolet–visible (UV–vis) spectroscopy, isothermal titration calorimetry (ITC). The association constants of this ternary complex were determined using isothermal titration calorimetry. The stability of the non-covalent interactions and self-assemblies form from this molecular recognition was confirmed by UV–vis spectroscopy and dynamic light scattering (DLS). A supramolecular nanomedicine was constructed on the basis of this 1:1:1 ternary recognition, and its in vitro and in vivo anticancer efficacy were thoroughly evaluated. Positron emission tomography (PET) imaging was used to monitor the delivery and biodistribution of the supramolecular nanomedicine.

**Results:**

Various experiments confirmed that the ternary complexation between 4,4′-bipyridinium, and napththyl derivative and CB[8] was stable in physiological environment, including phosphate buffered solution and cell culture medium. Supramolecular nanomedicine (SNM@DOX) encapsulating a neutral anticancer drug (doxrubincin, DOX) was prepared based on this molecular recognition that linked the hydrophobic poly(ε-caprolactone) chain and hydrophilic polyethylene glycol segment. The non-covalent interactions guaranteed the stability of SNM@DOX during blood circulation and promoted its tumor accumulation by taking advantage of the enhanced permeability and retention effect, thus greatly improving the anti-tumor efficacy as compared with the free drug.

**Conclusion:**

Arising from the host-enhanced charge-transfer interactions, the CB[8]-based ternary recognition was stable enough in physiological environment, which was suitable for the fabrication of supramolecular nanotheranostics showing promising potentials in precise cancer diagnosis and therapy.

**Graphic Abstract:**

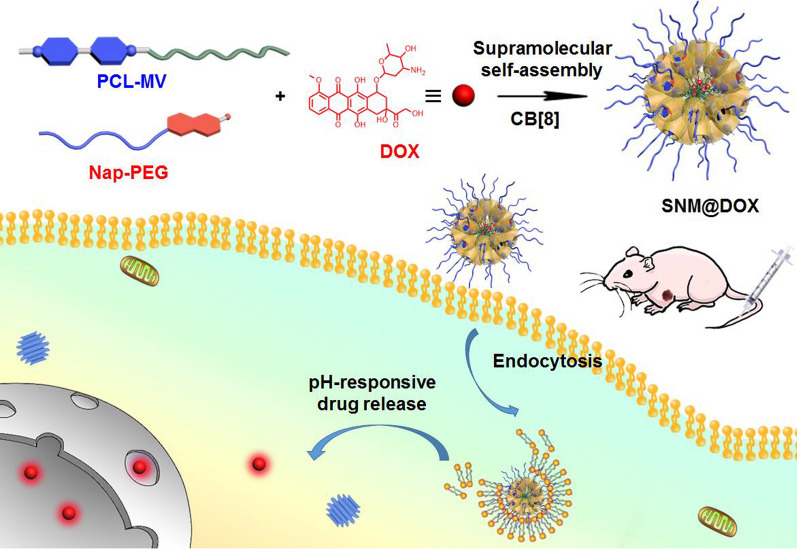

**Supplementary Information:**

The online version contains supplementary material available at 10.1186/s12951-021-01076-z.

## Introduction

Supramolecular theranostics combining diagnostic and therapeutic functions into one platform constructed from non-covalent interactions have attracted extensive attentions over the past years owing to their unparalleled advantages [1–4]. Imaging probes, therapeutic agents and targeting ligands can be integrated into the theranostic system through self-assembly on the basis of supramolecular chemistry, greatly avoiding tedious organic synthesis [5–7]. Additionally, the dynamic and reversible nature of non-covalent interactions endow the supramolecular theranostics with brilliant stimuli-responsiveness, activating the loaded drugs in sites of action triggered by the specific tumor microenvironment, possibly optimizing the theranostic performance and decreasing undesirable side effects [8–10]. Various non-covalent interactions have been employed to construct supramolecular theranostics, such as H-bonds, van der Waals forces, metal coordinations, electrostatic interactions and host–guest complexations [11, 12]. Attributing to their abundant responsivenesses to endogenous or exogenous stimuli, host–guest systems have gained numerous attentions in the development of supramolecular theranostics [13–17]. Macrocyclic hosts, like cyclodextrins, calixarenes, cucurbiturils, and pillararenes are widely utilized for the treatment of cancer and other diseases, which have achieved impressive outcomes [18–22]. Due to the differences in topological structures and substituents, the binding behaviors and affinities of these hosts with guest molecules are always distinct, which are extremely important for the fabrication of supramolecular theranostics.

Different from traditionally covalent bonds, non-covalent interactions show relatively weak affinities, which can be interfered by the external triggers in physiological environment. For example, H-bonds are remarkably impaired in aqueous solution by water molecules that act as H-bonds donors and acceptors. Electrostatic interactions and metal coordinations can be extremely weakened in the presence of cations, such as iron, copper, zinc *et c*, which largely exist in the body fluids. In the case of host–guest inclusion complexes, their interactions are sensitive to the salts, peptides, and proteins, which can also work as competitive molecules to interact with the hosts or guests. Considering the rigid structures and high association constants towards guests, cucurbiturils have been widely chosen for the construction of supramolecular polymers, sensors and drug delivery systems [23–33]. Especially for cucurbit[8]uril (CB[8]), its large cavity size (479 Å^3^) allows the formation of heteroternary complexes in the hollow cavity with a well-defined face-to-face π − π stacking geometry of the electron-rich donor and electron-deficient acceptor [34, 35]. This recognition motif is a practical donor − acceptor mix-and-match approach, the acceptor and donor molecules separately penetrates into the cavity as the first and second guest [36, 37]. The ability to form 1:1:1 ternary host−guest complexes distinguishes CB[8] from other macrocycles in the development of supramolecular nanomedicines, in which CB[8] acts as non-covalent linker to connect functional building blocks. Although this molecular recognition shows bright future in biomedical application, its stability in physiological environment is rarely evaluated.

**Scheme 1:**
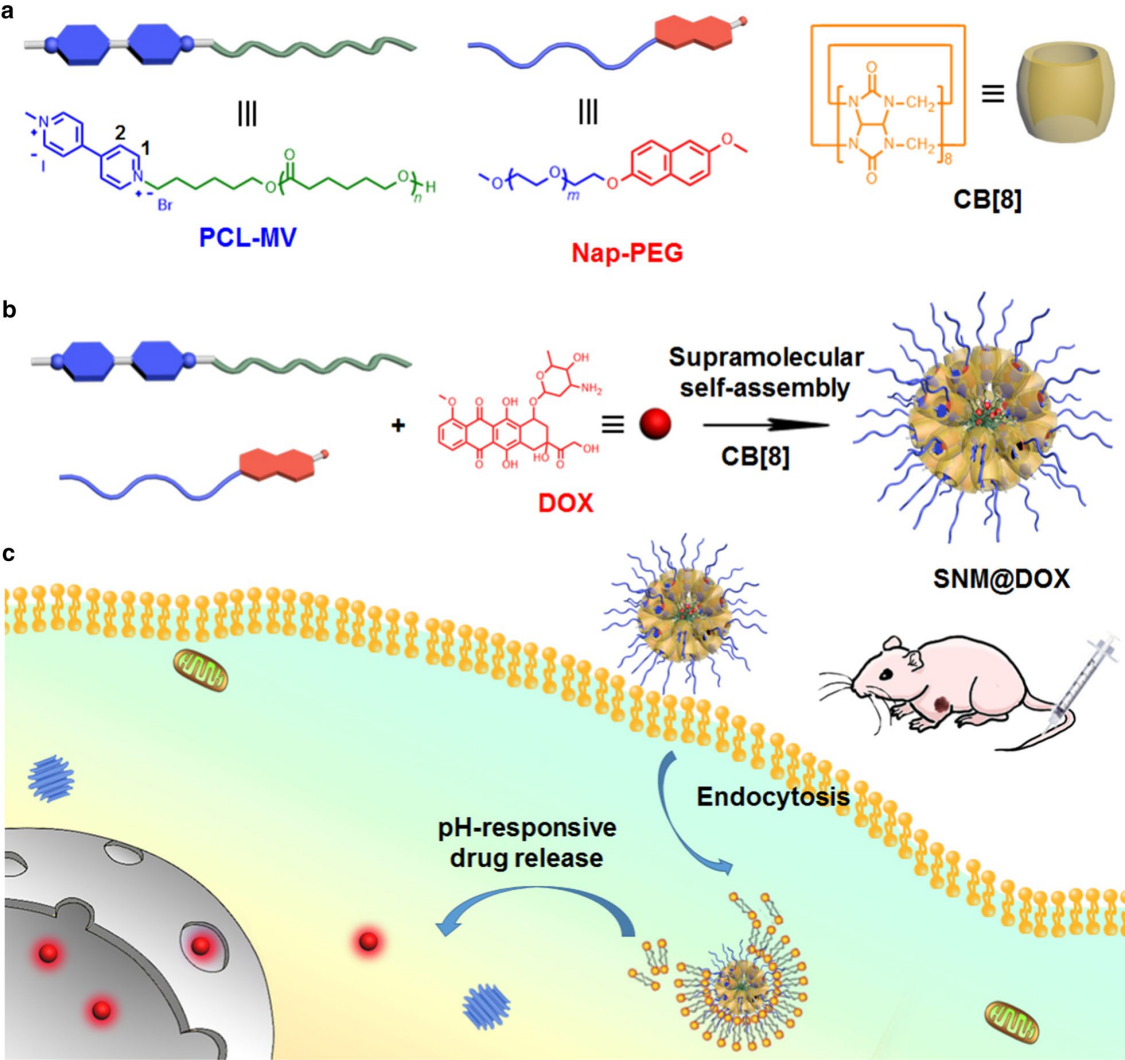
**a** The chemical structures of CB[8], Nap-PEG and PCL-MV. **b** The preparation of supramolecular nanomedicine SNM@DOX. **c** Schematic illustration of the drug delivery and cellular internalization of SNM@DOX.

Herein, we fully investigate the ternary host−guest complexation between CB[8], 4,4′-bipyridinium, and napththyl derivative, and determine the association constants in PBS and cell culture medium. The strong binding affinities ensure the heteroternary complexation in these physiological solutions, guaranteeing the stability of supramolecular delivery systems prepared from this host−guest recognition. Indeed, a supramolecular deblock copolymer is prepared using 4,4′-bipyridinium-modified poly(ε-caprolactone) (PCL-MV), 6-methoxy-2-naphthol-conjugated methoxy poly(ethylene glycol) (Nap-PEG) and CB[8] as building blocks (Scheme 1), which self-assembles into supramolecular nanoparticles (SNPs) in aqueous solution with the ability to encapsulate a hydrophobic anticancer drug DOX to afford a supramolecular nanomedicine (SNM@DOX). Benefiting from the strong binding affinities, SNM@DOX maintains intact in physiological environment, a pivotal role in the prevention of premature drug release. The supramolecular nanoformulation is readily labelled by a radioactive tracer (^89^Zr) through host−guest chemistry, monitoring its delivery in real time by PET imaging. Compared with free drug (doxorubicin hydrochloride, DOX∙HCl), the circulation time and tumor accumulation are greatly increased mainly attributing to the enhanced permeability and retention (EPR) effect. In vivo experiments demonstrate the excellent anti-tumor performance and low systemic toxicity of SNM@DOX.

## Results

### Characterizations of host−guest complexation

**Fig. 1:**
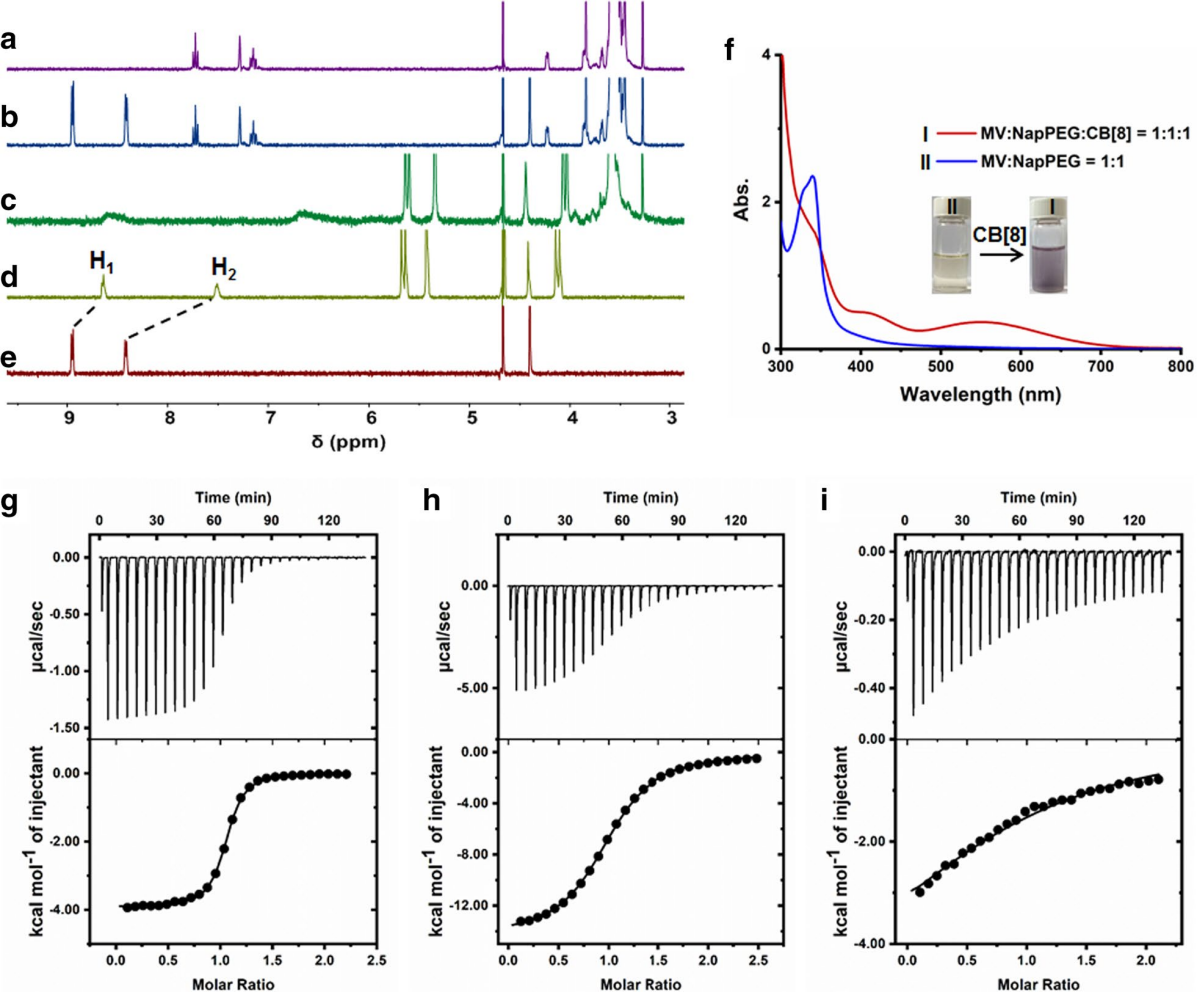
^1^H NMR spectra of **a** Nap-PEG, **b** Nap-PEG + MV, **c** Nap-PEG + MV +  CB[8], **d** CB[8] + MV, and **e** MV in D_2_O containing phosphate buffer (20.0 mM). **f** UV–vis spectra of the solution containing Nap-PEG (500 μM) and MV (500 μM) with/without of CB[8] (500 μM). **g** ITC data and fitting curve of MV (1.00 mM) titrated into CB[8] (0.100 mM) in phosphate buffer (20.0 mM) at 298.1 K. **h** ITC data and fitting curve of Nap-PEG (1.00 mM) titrated into the solotion of CB[8] (0.100 mM) and MV (0.100 mM) in phosphate buffer (20.0 mM) at 298.1 K. **i** ITC data and fitting curve of Nap-PEG (500 μM) titrated into the solotion of CB[8] (50.0 μM) and MV (50.0 μM) in DMEM cell culture medium at 298.1 K

Proton nuclear magnetic resonance (^1^H NMR) spectroscopy was firstly utilized to characterize the host −guest complexation using methylviologen (MV), and Nap-PEG as model guests (Fig. 1a − e). As shown in the ^1^H NMR spectra collected in PBS using D_2_O as a solvent, upfield shift changes were monitored for the signals related to the protons (H_1_ and H_2_) of MV upon addition of CB[8], indicating that the cationic guest deeply threaded into the cavity (Fig. 1d). The driving forces for the inclusion complexation benefited from the hydrophobic cavity and dipolar nature of the carbonyl-fringed portals of CB[8], which made the host highly attractive for dicationic guests where the cations were separated by a hydrophobic region through hydrophobic interactions and ion–dipole effect [35]. Notably, negligible changes in chemical shift were detected for peaks of Nap-PEG in the presence of MV (Fig. 1b), confirming negligible interactions between these two building blocks without CB[8]. Interestingly, obvious changes were observed upon addition of CB[8] into the solution containing Nap-PEG and MV (Fig. 1c). The peaks of the aromatic protons on Nap-PEG and MV disappeared due to the broaden effect, and the signal related to the protons of CB[8] shifted upfield, a convincing evidence for the formation of ternary host − guest complex. Host-enhanced charge-transfer (CT) interactions induced the formation of a 1:1:1 complex, because CB[8] possessed a large cavity that is able to encapsulate Nap-PEG and MV simultaneously, where MV acted as the electron acceptor and Nap-PEG acted as the electron donor.

Further evidence for the formation of CT interactions in the cavity of CB[8] came from UV–vis absorption spectroscopy (Fig. 1f). In sharp comparison with the mixture of Nap-PEG and MV in PBS, a broad absorption was measured ranging from 400 to 700 nm by adding CB[8] into the solution containing Nap-PEG and MV, corresponding to the typical CT band [36]. In addition, the solution color immediately turned into violet by mixing CB[8], Nap-PEG and MV (molar ratio = 1:1:1), a direct proof for the generation of a CT complex (Fig. 1f). The association constants (*K*_a_) of this ternary complex were determined by ITC, which provided the complexation thermodynamic behaviors and binding affinity. The *K*_1_ value between CB[8] and MV was calculated to be (1.53 ± 0.05) × 10^6^ M^−1^ with a 1:1 complexation stoichiometry in PBS (20.0 mM), verifying the strong interaction (Fig. 1g). By titration the solution of Nap-PEG into the solution of CB[8] and MV, *K*_2_ value was further calculated to be (1.54 ± 0.04) × 10^5^ M^−1^ (Fig. 1h), confirming the stepwise complexation. Thus, the final *K*_a_ value was about 2.36 × 10^11^ M^−2^, which demonstrated that the complexation was extremely stable in PBS. In order to assess the host−guest complexation in more complicated environment, the titration was conducted in cell culture medium containing fetal bovine serum, peptides and other substances, which possibly interfered the non-covalent interactions. As shown in Fig. 1i, the corresponding *K*_2_ value was measured to be (2.23 ± 0.17) × 10^4^ M^−1^ in Dulbecco's Modified Eagle Medium (DMEM). It should be noted that the solubility of CB[8] was extremely poor in DMEM, thus the *K*_1_ value could not be detected using ITC. By the formation of an inclusion complex with MV, the solubility of the formed complex was enough for ITC characterization. Compared with the *K*_2_ values in PBS, the binding affinity showed an obvious decrease in cell culture medium, but still maintained a high level, which could guarantee the complexation. These studies revealed that the 1:1:1 ternary complexation between CB[8], Nap-PEG and MV was stable in physiological environment, such as PBS and culture medium.

### Fabrication of supramolecular nanomedicine and in vitro studies

**Fig. 2:**
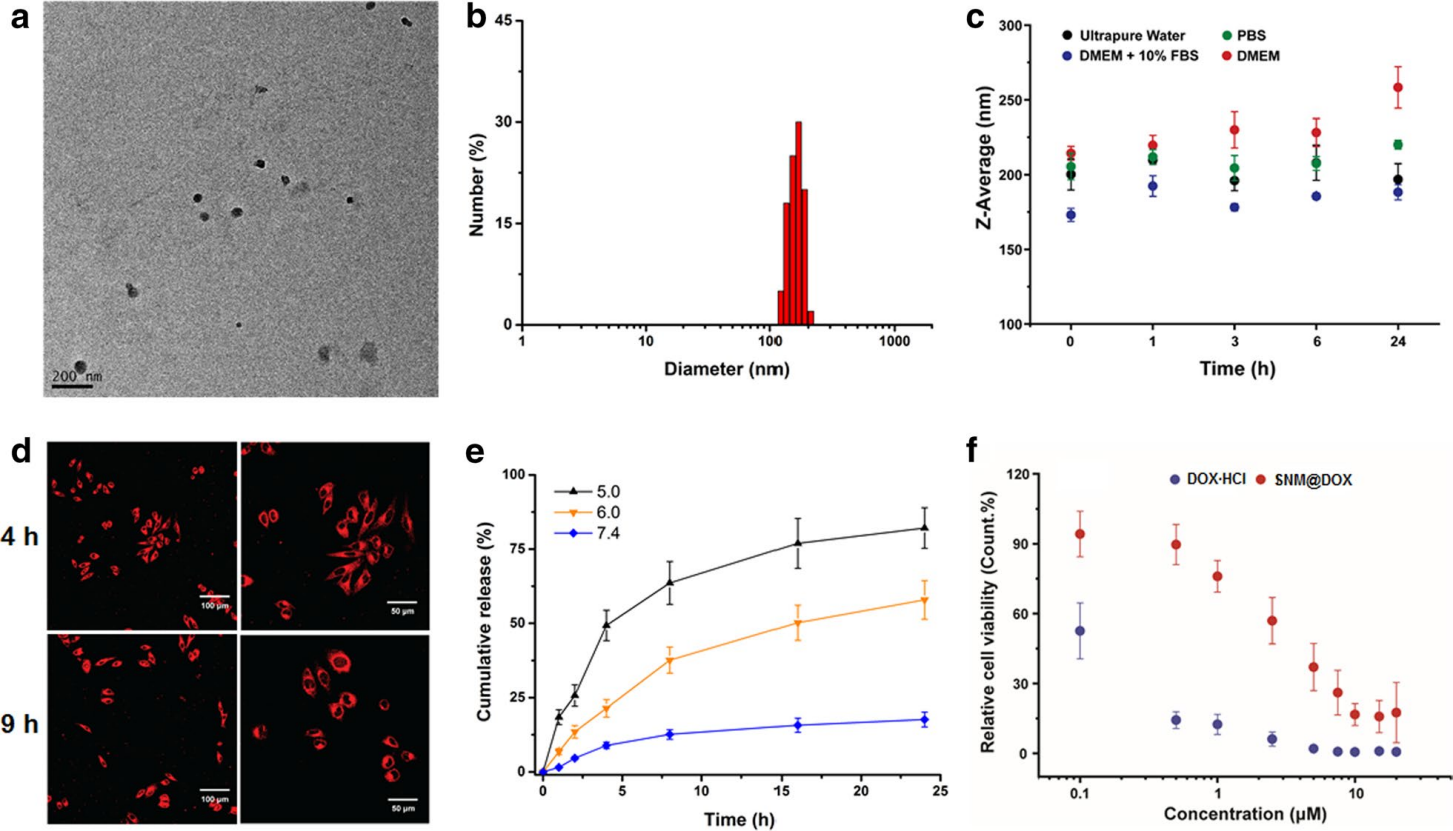
**a** TEM image of SNM@DOX. **b** DLS result of SNM@DOX in PBS. **c** The average diameters of SNM@DOX in different solutions. **d** CLSM images of HepG2 cells cultured with SNM@DOX for 4 and 9 h, respectively. The right images are the enlarged ones at the same time point. **e** Release profiles of DOX from SNM@DOX at different pH values. **f** Cytotoxicity of DOX∙HCl and SNM@DOX against HepG2 cells

With the molecular recognition in hand, we constructed a supramolecular copolymer using CB[8], Nap-PEG and PCL-MV, in which the hydrophobic and hydrophilic segments were linked by CB[8]. The amphiphilic nature of this supramolecular copolymer allowed to self-assemble into nanoparticles with a hydrophobic core to encapsulate a hydrophobic anticancer drug, like DOX. Indeed, a supramolecular nanomedicine (SNM@DOX) was successfully obtained with a drug loading content of 18.4%. Transmission electron microscopy (TEM) was used to reveal the size and morphology of SNM@DOX. Figure 2a indicated that nanoparticulate assemblies were found in TEM image 90 − 120 nm in diameter. The average diameter of SNM@DOX was determined to be 164.2 nm by dynamic light scattering (DLS), which was a little bigger than the size obtained from TEM because of the swelling effect in aqueous solution (Fig. 2b). Before in vitro and in vivo studies, the stability of SNM@DOX was assessed by DLS measurements in different solutions, including water, PBS, DMEM and DMEM + FBS, respectively. Figure 2c showed that negligible changes in mean diameter were detected, suggesting that SNM@DOX maintained stable in these solutions attributing to the strong binding affinity of this host−guest molecular recognition, ensuring the biomedical applications of SNM@DOX.

Confocal laser scanning microscopy (CLSM) was utilized to reveal the cellular uptake of SNM@DOX (Fig. 2d), which showed bright red signal in cytoplasma after 4 h incubation. The intracellular fluorescence of DOX became more intensive and some signal appeared in the nucleus by extending the culture time to 9 h. It should be emphasized that the endocytosis pathway of SNM@DOX was different from that of free DOX·HCl (Additional file 1: Fig. S17), which translocated into nucleus immediately after cellular internalization. DOX containing an amine group can be protonated in acidic environment, thus accelerating its release from the nanomedicine. To mimic the pH gradient from blood stream to the endo/lysosome, release behaviors of DOX from SNM@DOX were carried out at pH 7.4, 6.0, and 5.0, respectively (Fig. 2e). SNM@DOX kept stable in PBS at pH 7.4, only small amount (17.6%) of DOX released from SNM@DOX within 24 h incubation. The release rate and amount speed up effectively at low pH value, 57.9% of DOX was released from SNM@DOX at pH 6.0 and 82.1% at pH 5.0 within the same period, respectively.

A 3-(4',5'-dimethylthiazol-2'-yl)-2,5-diphenyl tetrazolium bromide (MTT) assay was then chosen to evaluate the cytotoxicity of the delivery vehicles. Little influence on cell viability of CB[8], Nap-PEG and PCL-MV even at a relatively high concentration (Additional file 1: Fig. S18 − 20), an indicator of excellent biocompatibility. The anticancer efficacy of SNM@DOX against HepG2 cancer cells was assessed by CCK-8 assay. The half maximal inhibitory concentration (IC_50_) values of SNM@DOX were determined to be 10.7 ± 1.14 and 3.51 ± 0.41 μM after 24 and 48 h incubation, respectively (Fig. 2f, Additional file 1: Fig. S21). Compared with the free drug, the toxicity of DOX attenuated after nanoformulation mainly ascribing to the changes in endocytosis pathway and time-dependent release inside cells, while the cytotoxicity evaluation also elucidated that SNM@DOX kept satisfactory anticancer capability.

### In vivo anti-tumor performances

**Fig. 3:**
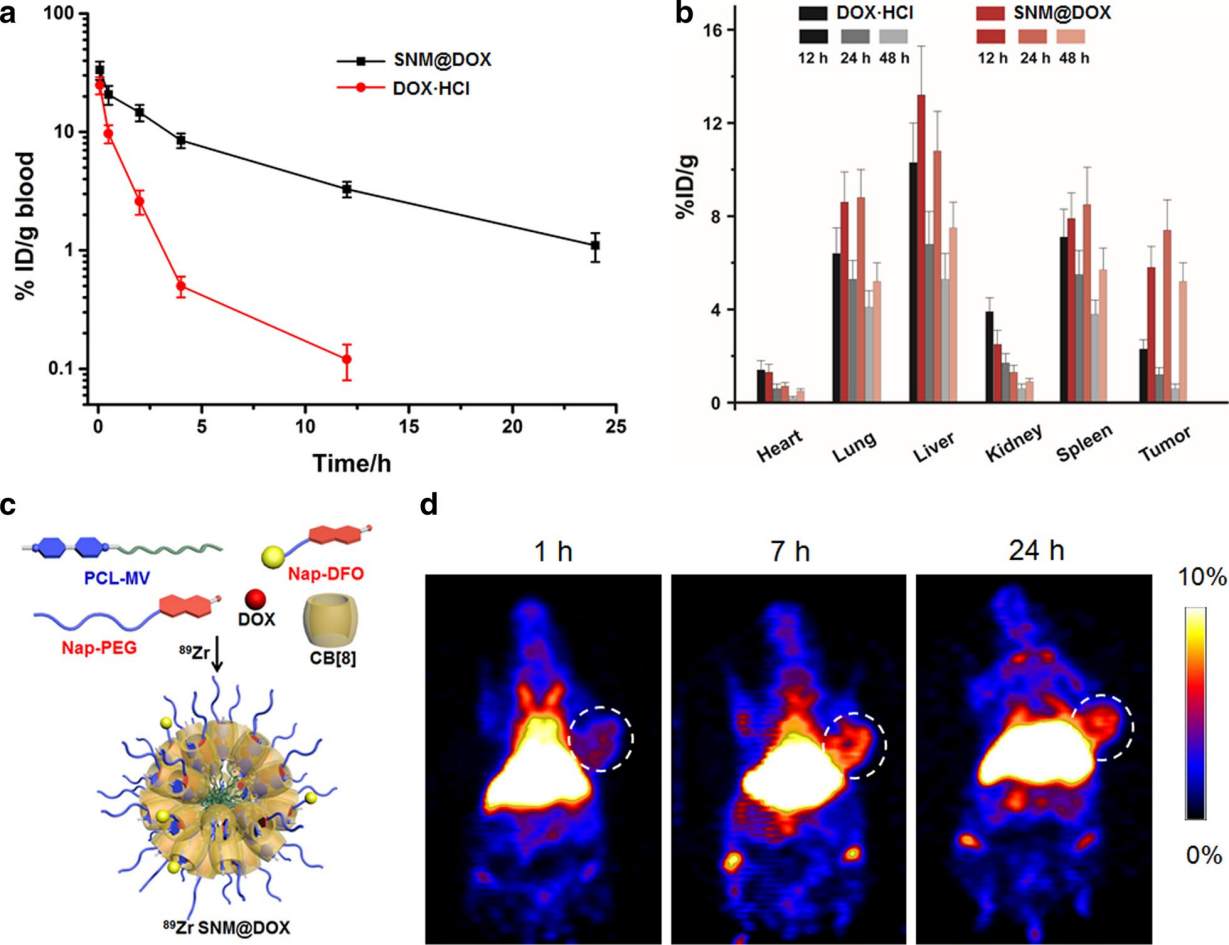
**a** Plasma concentration of DOX·HCl and SNM@DOX versus time after *i.v*. injection. **b** Time-dependent biodistributions of DOX·HCl and SNM@DOX in the main organs. **c** Schematic illustration of the preparation of ^89^Zr SNM@DOX through multi-components self-assembly followed by radiolabelling. **d** PET images of the mice bearing HepG2 tumor at different time post *i.v*. injection of ^89^Zr SNM@DOX

The pharmacokinetic behaviors and time-dependent biodistributions of SNM@DOX were investigated before in vivo anti-tumor treatment, and free DOX·HCl was chosen as a control. After intravenous (*i.v*.) injection, the blood was collected at different time post injections. By plotting the drug amount in blood versus injection time, the circulation half-life could be obtained. DOX·HCl was cleared out from body rapidly with a half-life of 0.30 ± 0.03 h, while the circulation half-life of SNM@DOX prolonged to 1.47 ± 0.16 h after nanoformulation benefiting from the EPR effect (Fig. 3a). The area under the curve of SNM@DOX was also much larger than that of DOX·HCl, which provided the possibility of enhanced drug accumulation in the tumor. The preferred tumor accumulation of SNM@DOX was supported by time-dependent biodistribution analysis. The intratumoral amount of SNM@DOX was 5.82 ± 0.86%ID/g at 12 h post *i.v*. injection, which increased to 7.41 ± 0.92%ID/g and 5.24 ± 0.78%ID/g at 24 h and 48 h post injection, respectively (Fig. 3b). The “brushlike” PEG shell of SNM@DOX prevented protein adsorption that was favorable to prolong blood circulation time, facilitating the accumulation of SNM@DOX in tumor sites [36–40]. In contrast, the tumor accumulation of DOX·HCl was much lower, the highest amount in tumor was 2.33 ± 0.42%ID/g occurred at 12 h post injection, and the drug eliminated from body quickly (Fig. 3b).

As a highly sensitive and noninvasive nuclear imaging technique, PET is widely used as a diagnostic molecular imaging tool by clinicians for preclinical and clinical imaging. By administrating a small amount of radiotracer, PET imaging can provide quantitative readout of pharmacokinetics, organ/tissue efficiency and tumor targeting efficiency in vivo, facilitating drug discovery and radiopharmaceutical development. A chelator Nap-DFO was obtained through three-step synthesis, which could chelate radioactive ^89^Zr and further modified the supramolecular nanoparticles through host−guest complexation to afford ^89^Zr SNM@DOX for PET imaging (Fig. 3c). The mice bearing HepG2 tumor were intravenously injected with ^89^Zr SNM@DOX imaged at various time points. The dynamic biodistributions and accumulations in main organs could be clearly visualized in the whole-body PET images shown in Additional file 1: Fig. S23, such as liver and tumor. The prolonged circulation time of the supramolecular nanomedicine was also confirmed by PET imaging, in which intensive signal was observed in heart at 1 h post injection. Intriguingly, ^89^Zr SNM@DOX exhibited an efficient accumulation in tumor sites in a time-dependent manner after injection. Quantitative analysis in the region-of-interest of these images coincided with the results in Fig. 3d, the intra-tumoral amount of ^89^Zr SNM@DOX was 0.92% ID g^–1^ at 1 h post injection, which increased to 4.14 and 6.52% ID/g at 7 and 24 h post injection, respectively.

**Fig. 4:**
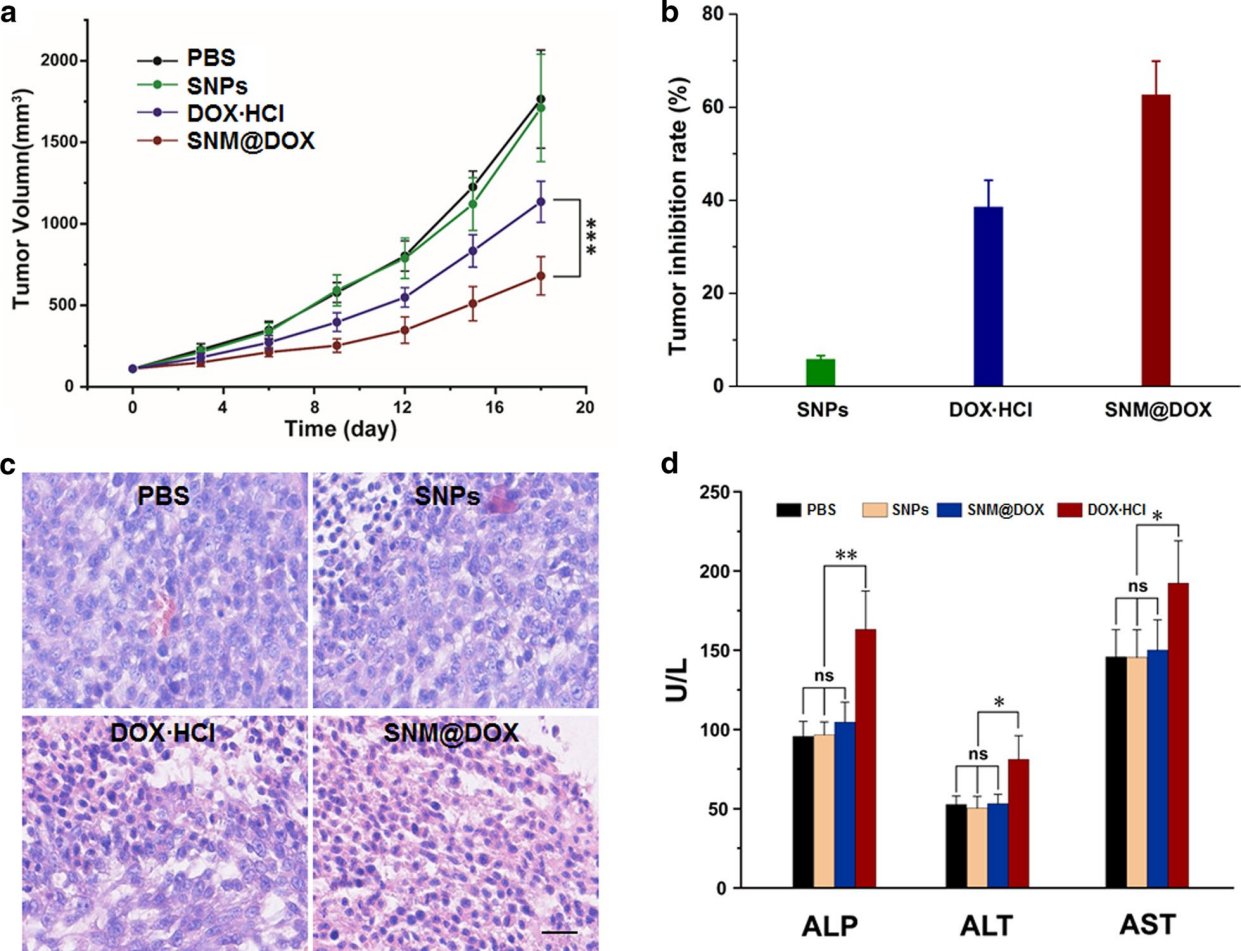
**a** Tumor volume changes of the mice administrated with different formulations. ***p < 0.001. **b** Tumor inhibition rate of the mice administrated with different formulations. **c** H&E staining of the tumor tissues from the mice treated with PBS, SNPs, DOX·HCl, and SNM@DOX, respectively. Scale bar is 50 μm. **d** Blood biochemistry tests of ALP, AST, and ALT from the mice treated with different formulations. *p < 0.05, **p < 0.01

In vivo anti-tumor ability of SNM@DOX was evaluated using HepG2 tumor-bearing mice that were intravenously injection with PBS, SNPs, free DOX·HCl, and SNM@DOX, respectively. The tumors grew rapidly for the mice treated with PBS or SNPs, the average tumor volume respectively increased to 912 and 1155 mm^3^ after 18 days (Fig. 4a), suggesting that the delivery vehicles had no influence on the tumor inhibition. Compared with the control mice treated with PBS, the formulation of free DOX·HCl resulted in a moderate tumor inhibition mainly attributing to the poor tumor accumulation and fast blood clearance, the inhibition rate was calculated to be 38.5% at the end day of therapy. In marked contrast, the tumor growth was effectively delayed for the mice administrated with SNM@DOX with a tumor inhibition rate of 62.6% (Fig. 4b), demonstrating the high anti-tumor efficacy of SNM@DOX treatment. The excellent therapeutic performance was further verified by hematoxylin and eosin (H&E) staining, in which highest level of apoptotic and necrotic cancer cells were found characterized by nuclear shrinkage and fragmentation for the mice treated with SNM@DOX (Fig. 4c).

Body weight changes were carefully monitored during therapeutic period to assess the systemic toxicity. The body weight decreased obviously at the firstly ten days for the mice administrated with DOX·HCl because of its side effects (Additional file 1: Fig. S24). The systemic toxicity of the loaded drug greatly relieved by the formation of nanoformulation, negligible changes in body weight was observed during therapy for mice injected with SNM@DOX. The clinical use of DOX and other quinone-hydroquinone anticancer anthracyclines is limited by a dose-related cardiotoxicity, a leading factor for drug withdrawals. Compared with healthy mice, histological analysis of heart tissue indicated that no obvious tissue lesions were detected for the mice injected with SNM@DOX (Additional file 1: Fig. S25). Moreover, the hepatotoxicity of the mice treated with DOX·HCl or SNM@DOX was assessed by detecting the level of alkaline phosphatase (ALP), aspartate aminotransferase (AST), and alanine aminotransferase (ALT) in blood (Fig. 4d). An apparent elevation of these parameters was found for the mice receive free DOX·HCl, while these indicators were normal for the mice injected with SNM@DOX when compared with the healthy mice. Benefiting from the EPR effect and high stability of SNM@DOX, undesirable adverse reactions of the anticancer drug were successfully attenuated through this supramolecular formulation.

## Conclusions

In conclusion, the ternary host−guest complexation between MV, napththyl derivative and CB[8] in physiological environment was fully investigated. ^1^H NMR, ITC and UV–vis measurements collectively demonstrated that this ternary molecular recognition was stable in PBS and even cell culture medium containing FBS. The strong binding affinity in these aqueous solutions guaranteed the stability of supramolecular assemblies prepared from this recognition, which was extremely important for biomedical applications. A supramolecular diblock copolymer was constructed based on this 1:1:1 ternary complexation, which was further utilized to encapsulate a hydrophobic anticancer drug to afford a supramolecular nanomedicine SNM@DOX. Owing to the EPR effect, sophisticated engineering and non-covalent interactions, SNM@DOX possessed several advantages, such as high blood stability, prolonged circulation half-life, and abundant tumor accumulation. The delivery, biodistributions and accumulations of SNM@DOX in main organs were captured in real time through PET imaging by fully exploiting the host−guest complexation using a napththyl-modified chelator Nap-DFO. In vivo studies demonstrated the excellent therapeutic efficacy and attenuated systemic toxicity of SNM@DOX arising from the supramolecular nanoformulations. This pioneering work paves the way for the fabrication of supramolecular theranostics with promissing potentials in clinical trials on the basis of non-covalent interactions.

## Materials and methods

1,5,7-triazabicyclo[4.4.0]dec-5-ene (TBD), *ε*-caprolactone, 6-bromo-1-hexanol, *p*-toluenesulfonyl chloride, methoxyl polyethylene glycol (mPEG-OH, *M*_w_ = 2 kDa) were purchased from Sigma–Aldrich. Solvents were either employed as purchased or dried according to procedures described in the literature. Millipore ultrapure water was obtained on a Milli-Q purification system. Transmission electron microscopy (TEM) investigations were carried out on a HT-7700 instrument. UV–vis absorption spectra were recorded by using a Hitachi U-3010 spectrophotometer. Confocal laser scanning microscopy (CLSM) images was recorded on a LSM710META (Zeiss) microscope. Gel permeation chromatography (GPC) was conducted on a Waters Chromatography, Inc. (Milford, MA) system using THF containing 0.05 M LiBr as eluent. The sizes of the nanoformulations were determined by a DLS analyzer (Zetasizer Nano ZS90 Malvern Instruments, Malvern) with a detection angle of 90° at 25 °C using an incident He–Ne laser (λ = 633 nm). ITC experiments were carried out with a Microcal VP-ITC calorimeter at 298.1 K. The anticancer efficacy was determined by using Cell Counting Kit-8 (CCK-8, Solarbio, Beijing) according to the instructions of the manufacturer. The absorbance of the bioreduced soluble formazan product was measured at 450 nm using a TECAN Infinite F200 PRO. H&E tissue and cell staining was performed by BBC Biochemical (Mount Vernon, WA) and the images were collected using a BX41 bright field microscopy (Olympus).

### Synthesis of PCL-MV

The synthetic routes of PCL-MV and Nap-PEG were illustrated in Additional file 1: Scheme S1. To a solution of 6-bromo-1-hexanol (181 mg, 1.00 mmol) and *ε*-caprolactone (4.56 g, 40.0 mmol) in anhydrous CH_2_Cl_2_ (10 mL), TBD (139 mg, 1.00 mmol) was added and the mixture was stirred at room temperature. After 20 min, the reaction was quenched by adding 1 mL of acetic acid. The resulting solution was precipitated into an excess of diethyl ether. After filtration, the sediments was dissolved in CH_2_Cl_2_ and precipitated into an excess of diethyl ether; the above dissolution–precipitation cycle was repeated three times. After drying in a vacuum oven overnight at room temperature, PCL-Br was obtained as a white solid (4.45 g, yield: 93.9%). The molecular weight and composition of PCL-Br were determined by ^1^H NMR spectroscopy (Additional file 1: Fig. S1) and GPC (Additional file 1: Fig. S2).

A mixture of PCL-Br (1.15 g) and excessive MVI (700 mg) in DMF (10 mL) was stirred at 85 °C overnight. After reaction, the solvent was poured into 150 mL of H_2_Cl_2_, and the mixture was washed with water for three times. The solvent was evaporated and the residue was dissolved in 10 mL of THF. The resulting solution was precipitated into an excess of diethyl ether. The above dissolution–precipitation cycle was repeated three times. The solid was dried overnight in a vacuum to give a pale yellow powder with a yield of 92.7%. The molecular weight and composition of PCL-MV were determined by ^1^H NMR spectroscopy (Additional file 1: Fig. S3) and GPC (Additional file 1: Fig. S4).

### Synthesis of Nap-PEG

mPEG-OH (10.0 g, 5.00 mmol) and NaOH (4.00 g, 100 mmol) were dissolved in the mixture of THF and H_2_O (150 mL, THF/H_2_O = 1/1, *v*/*v*). *p*-Toluenesulfonyl chloride (5.70 g, 30.0 mmol) was dissolved in 30 ml of THF, and the solution was droply added into the mPEG-OH solution at 0 °C. The mixture was further stirred at room temperature overnight. The organic solvent was evaporated, and the solution was extracted by CH_2_Cl_2_ (3 × 50 mL). The organic phase was further washed with water for three times to eliminate the excessive NaOH and *p*-toluenesulfonyl chloride. The solvent was concentrated into 10 mL, and the resulting solution was precipitated into an excess of diethyl ether. The above dissolution–precipitation cycle was repeated three times to afford mPEG-OTs without further purification.

A mixture containing mPEG-OTs (2.15 g), 6-methoxy-2-naphthol (1.74 g, 10.0 mmol) and K_2_CO_3_ (2.76 g, 20.0 mmol) in CH_3_CN (50 mL) was added to a round-bottom flask under nitrogen atmosphere and heated at reflux for 12 h. The organic phase was obtained after filtration and the solvent was removed by rotary evaporation to afford the crude product. The residue was dissolved in 5 mL of CH_2_Cl_2_, and the resulting solution was precipitated into an excess of diethyl ether. The above dissolution–precipitation cycle was repeated three times. The solid was dried overnight in a vacuum to give a dark green powder with a yield of 87.4%. The molecular weight and composition of PCL-MV were determined by ^1^H NMR spectroscopy (Additional file 1: Fig. S5) and GPC (Additional file 1: Fig. S6).

### Synthesis of Nap-DFO

The synthetic route of Nap-DFO was illustrated in Additional file 1: Scheme S2. A mixture containing tert-butyl *N*-(2-bromoethyl)carbamate (2.24 g, 10 mmol), 6-methoxy-2-naphthol (0.87 g, 5.00 mmol) and K_2_CO_3_ (2.76 g, 20.0 mmol) in CH_3_CN (50 mL) was added to a round-bottom flask under nitrogen atmosphere and heated at reflux for 12 h. The organic phase was obtained after filtration and the solvent was removed by rotary evaporation to afford the crude product, which was isolated by flash column chromatography to give Nap-Boc as a gray solid with a yield of 76.8%. ^1^H NMR (Additional file 1: Fig. S7), ^13^C NMR (Additional file 1: Fig. S8) and mass (Additional file 1: Fig. S9) spectra were utilized to confirm the preparation of Nap-Boc.

Trifluoroacetic acid (2.00 mL) was added to the solution of Nap-Boc (0.63 g, 2 mmol) in CH_2_Cl_2_ (15 mL), and the mixture was stirred at room temperature for 8 h. The solvent was removed by rotary evaporation and the compound was washed by methanol for three times to give Nap-NH_2_ as a brown solid with a yield of 64.5%.^1^H NMR (Additional file 1: Fig. S10), ^13^C NMR (Additional file 1: Fig. S11) and mass (Additional file 1: Fig. S12) spectra were utilized to confirm the preparation of Nap-NH_2_. Nap-DFO was obtained by labelling Nap-NH_2_ with NCS-DFO, and the successful preparation of Nap-DFO was verified by ^1^H NMR (Additional file 1: Fig. S13), ^13^C NMR (Additional file 1: Fig. S14) and mass spectrum (Additional file 1: Fig. S15).

### Preparation of supramolecular nanomedicine

DOX (8.00 mg), PCL-MV (18.5 mg) and Nap-PEG (11.2 mg) were dissolved in DMSO (10 mL), 10 mL of aqueous solution containing CB[8] (1.00 mg/mL) was droply added into the mixture solution. After stirring in the dark for 2 h, the resulting mixture was sealed in dialysis bags with a molecular weight cut-off of 3.5 kDa and dialyzed against DI water for 12 h to remove free DOX and CB[8]. The drug loading content was estimated to be 18.4% by using UV spectroscopy. For the preparation of ^89^Zr labelled nanomedicine, DOX (8.00 mg), PCL-MV (18.5 mg), Nap-DFO (0.300 mg), and Nap-PEG (10.5 mg) were dissolved in DMSO (10 mL), 10 mL of aqueous solution containing CB[8] (1.00 mg/mL) was droply added into the mixture solution. After stirring in the dark for 2 h, the resulting mixture was sealed in dialysis bags with a molecular weight cut-off of 3.5 kDa and dialyzed against DI water for 12 h to remove Nap-DFO, free DOX and CB[8]. SNM@DOX was labelled with ^89^Zr by mixing the nanomedicine with radioactive isotope at 37 °C for 1 h under constant stirring.

### Drug release studies

In vitro released profiles of DOX from SNM@DOX at different pH value were monitored using the dialysis method. The SNM@DOX was dissolved in 25 mL of distilled water and sealed in dialysis bags with a molecular weight cut-off of 2 kDa at pH 7.4, 6.0, and 5.0, respectively. The dialysis apparatus was agitated on an orbital shaker at 100 rpm at 37 °C. At designated time intervals, 1 mL of medium was taken out from the 25 mL solution out of the dialysis bag for UV detection and was then put back to the original system. The DOX concentration was calculated with a standard curve calibrated with DOX samples of known concentrations.

### Cell cultures

HepG2 cells were cultured in Dulbecco's modified Eagle's medium (DMEM) containing 10% fetal bovine serum (FBS) and 1% penicillin/streptomycin. Cells grew as a monolayer and were detached upon confluence using trypsin (0.5% *w*/*v* in PBS). The cells were harvested from the cell culture medium by incubating in a trypsin solution for 5 min. The cells were centrifuged, and the supernatant was discarded. A 3 mL portion of serum-supplemented DMEM was added to neutralize any residual trypsin. The cells were resuspended in serum-supplemented DMEM at a concentration of 1 × 10^4^ cells/mL. Cells were cultured at 37 °C and 5% CO_2_.

### Evaluation of cytotoxicity

The cytotoxicities of CB[8], PCL-MV, Nap-PEG, DOX·HCl, and SNM@DOX against HepG2 cells were determined by MTT or CCK-8 assay in a 96-well cell culture plate. All solutions were sterilized by filtration with a 0.22 μm filter before tests. HepG2 cells were seeded at a density of 1 × 10^4^ cells/well in a 96-well plate, and incubated for 24 h for attachment. Cells were then incubated with CB[8], PCL-MV, Nap-PEG, DOX·HCl, and SNM@DOX at various concentrations for 24 h. After washing the cells with PBS buffer, 20 μL of a MTT solution (5 mg/mL) was added to each well. After 4 h of incubation at 37 °C, the MTT solution was removed, and the insoluble formazan crystals that formed were dissolved in 100 μL of dimethylsulfoxide (DMSO). The absorbance of the formazan product was measured at 570 nm using a spectrophotometer (Bio-Rad Model 680). For CCK-8 assay, WST-8 [2-(2-methoxy-4-nitrophenyl)-3-(4-nitrophenyl)-5-(2, 4-disulfophenyl)-2H-tetrazolium, monosodium salt] solution was added and cells were incubated for 4 h at 37 °C under 5% CO2. The absorbance of each well was measured with a luminescence microplate reader (Bio-Rad 680) at 450 nm. Untreated cells in media were used as a control. All experiments were carried out with five replicates.

### Cellular internalization studies by CLSM

HepG2 cells were treated with SNM@DOX (the concentration of DOX was 2.00 μM) in the culture medium at 37 °C for 4 and 9 h, respectively. The cells were washed three times with PBS and fixed with fresh 4.0% formaldehyde at room temperature for 15 min. After washing with PBS, the cells were stained with DAPI (1 μg/mL) for 15 min. The images were taken using a LSM-510 confocal laser scanning microscope (Zeiss, Germany) (100 × oil objective, 405/488 nm excitation).

### Animals and tumor models

Female nude mice (4 weeks old, ~ 20 g body weight) were purchased from Zhejiang Academy of Medical Sciences and maintained in a pathogen-free environment under controlled temperature (24 °C). Animal care and handling procedures were in agreement with the guidelines evaluated and approved by the ethics committee of Zhejiang University of Technology. Study protocols involving animals were approved by the Zhejiang University of Technology Animal Care and Use Committee. The female nude mice were injected subcutaneously in the right flank region with 200 μL of cell suspension containing 2 × 10^6^ HepG2 cells. The tumors were allowed to grow to ~ 100 mm^3^ before experimentation. The tumor volume was calculated as (tumor length) × (tumor width)^2^/2. Relative tumor volumes were calculated as *V*/*V*_0_ (*V*_0_ was the tumor volume when the treatment was initiated).

### Pharmacokinetics and biodistribution

For pharmacokinetic studies, the mice were randomly divided into two groups (*n* = 3). The aqueous solutions of free DOX·HCl, and SNM@DOX were *i.v.* injected via tail vein at a dose of 10.0 mg DOX/kg. The blood samples (0.1 mL) were taken from the eye socket at the different time points post injection. The plasma was obtained by centrifugation at 3000 rpm for 15 min, and the amount of DOX in the plasma was assayed by HPLC. The DOX concentrations in the tumor tissues and organs were analyzed by HPLC. 200 μL of 10% (*w*/*v*) tissue homogenate was added with 100 μL of PBS. The above mixture was subsequently extracted with chloroform/isopropanol (4:1, *v*/*v*) by vortex mixing for 3 min. After centrifugation at 10,000 rpm for 5 min, the organic phase was separated and evaporated to dryness under a stream of nitrogen. The residue was dissolved in 200 μL of mobile phase (methanol/water/acetic acid = 65:35:2, *v*/*v*/*v*). After centrifugation at 10,000 rpm for 5 min, the supernatant was collected for HPLC analysis. The mice bearing HepG2 tumors were *i.v*. injected ^89^Zr SNM@DOX and anesthetized with isoflurane (1.0 ~ 2.0%) in oxygen delivered at a flow rate of 1.0 L/min. All PET imaging scans were conducted on a micro-PET/CT at different time post injection.

### In vivo anti-tumor evaluation

The mice were divided into three treatment groups randomly (*n* = 5), when the mean tumor volume reached about 100 mm^3^ and this day was set as day 0. Mice were administered intravenously with PBS, SNPs, free DOX·HCl (5.00 mg DOX/kg), and SNM@DOX (5.00 mg DOX/kg), respectively every 3 days for four times. Tumor volume and body weight were measured every 3 days. The tumor inhibition study was stopped on the 18th day. In the histological assay, the tissues were fixed in 4% paraformaldehyde for 24 h. The specimens were dehydrated in graded ethanol, embedded in paraffin, and cut into 5 mm thick sections. The fixed sections were deparaffinized and hydrated according to a standard protocol and stained with hematoxylin and eosin (H&E) for microscopic observation.

### Statistical analysis

Data are presented as the mean ± standard deviation. Statistical analysis of data was performed with one-way analysis of variance. The level of significance was defined at *p < 0.05, **p < 0.01, ***p < 0.001.

## Supplementary material

Experimental details and supporting data. This material is available free of charge via the Internet at https://jnanobiotechnology.biomedcentral.com/.

## Supplementary Information

**Additional file 1: Scheme S1**. Synthetic routes to PCL-MV and Nap-PEG. **Scheme S2**. Synthetic routes to Nap-DFO. **Fig. S1**. ^1^H NMR spectrum (400 MHz, CDCl3, room temperature) of PCL-Br. **Fig. S2**. GPC curve of PCL-Br. **Fig. S3**. ^1^H NMR spectrum (400 MHz, CDCl3, room temperature) of PCL-MV. **Fig. S4**. GPC curve of PCL-MV. **Fig. S5**. ^1^H NMR spectrum (400 MHz, CDCl3, room temperature) of Nap-PEG. **Fig. S6**. GPC curve of Nap-PEG. **Fig. S7**. ^1^H NMR spectrum (400 MHz, CDCl3, room temperature) of Nap-Boc. **Fig. S8**. 13C NMR spectrum (100 MHz, CDCl3, room temperature) of Nap-Boc. **Fig. S9**. ESI IT-TOF results of Nap-Boc. [M + Na]+ = 340.1494. **Fig. S10**. ^1^H NMR spectrum (400 MHz, DMSO-d6, room temperature) of Nap-NH2. **Fig. S11**. ^13^C NMR spectrum (100 MHz, DMSO-d6, room temperature) of Nap-NH2. **Fig. S12**. ESI IT-TOF results of Nap-NH2. [M + H]+ = 218.1142. **Fig. S13**. ^1^H NMR spectrum (400 MHz, DMSO-d6, room temperature) of Nap-DFO. **Fig. S14**. ^13^C NMR spectrum (100 MHz, DMSO-d6, room temperature) of Nap-DFO. **Fig. S15**. ESI IT-TOF result of Nap-DFO. [M – H]– = 775.3225. **Fig. S16**. ^1^H NMR spectra of a Nap-PEG, b Nap-PEG + CB[8] and c CB[8] in D2O. **Fig. S17**. CLSM images of HepG2 cells cultured with DOX∙HCl for 4 h and 9 h, respectively. The right images are the enlarged ones at the same time point. **Fig. S18**. Cytotoxicity evaluation of CB[8] against HepG2 cells using an MTT assay. **Fig. S19**. Cytotoxicity evaluation of PCL-MV against HepG2 cells using an MTT assay. **Fig. S20**. Cytotoxicity evaluation of Nap-PEG against HepG2 cells using an MTT assay. **Fig. S21**. Cytotoxicity evaluation of DOX·HCl and SNM@DOX against HepG2 cells after 24 h incubation using a CCK-8 assay. **Fig. S22**. Release profiles of Nap-DFO from the nanoformulation in water or PBS. **Fig. S23**. Time-dependent biodistribution of 89Zr SNM@DOX in liver and tumor. **Fig. S24**. Body weight changes of the mice treated with different formulations. **Fig. S25**. H&E staining of the heart tissues from a healthy mouse and b the mouse treated with SNM@DOX at day 18.

## Data Availability

The datasets generated and/or analyzed during the current study are available from the corresponding authors on reasonable request.

## Abbreviations

CB[8]: Cucurbit[8]uril
ITC: Isothermal titration calorimetry
DOX: Doxrubincin
PEG: Polyethylene glycol
PCL: Poly(ε-caprolactone)
EPR: Enhanced permeability and retention
PBS: Phosphate bufer saline
CT: Charge-transfer
*K*_a_: Association constants
TEM: Transmission electron microscopy
%ID/g: Injected dose per gram
DLS: Dynamic light scattering
CLSM: Confocal laser scanning microscopy
MTT: 3-(4′,5′-Dimethylthiazol-2′-yl)-2,5-diphenyl tetrazolium bromide
IC_50_: Half maximal inhibitory concentration
PET: Positron emission tomography
H&E: Hematoxylin and eosin
ALT: Alanine amino transferase
AST: Aspartate aminotransferase
ALP: Alkaline phosphatase
