# Supplementary material for: Evaluation of the stability of cucurbit[8]uril-based ternary host−guest complexation in physiological environment and the fabrication of a supramolecular theranostic nanomedicine
Source: J Nanobiotechnology. 2021 Oct 20;19:330. doi: 10.1186/s12951-021-01076-z (PMC8529793; doi:10.1186/s12951-021-01076-z)
Supplement: Supplementary file 1 — Additional file 1: Scheme S1. Synthetic routes to PCL-MV and Nap-PEG. Scheme S2. Synthetic routes to Nap-DFO. Fig. S1. 1H NMR spectrum (400 MHz, CDCl3, room temperature) of PCL-Br. Fig. S2. GPC curve of PCL-Br. Fig. S3. 1H NMR spectrum (400 MHz, CDCl3, room temperature) of PCL-MV. Fig. S4. GPC curve of PCL-MV. Fig. S5. 1H NMR spectrum (400 MHz, CDCl3, room temperature) of Nap-PEG. Fig. S6. GPC curve of Nap-PEG. Fig. S7. 1H NMR spectrum (400 MHz, CDCl3, room temperature) of Nap-Boc. Fig. S8. 13C NMR spectrum (100 MHz, CDCl3, room temperature) of Nap-Boc. Fig. S9. ESI IT-TOF results of Nap-Boc. [M + Na]+ = 340.1494. Fig. S10. 1H NMR spectrum (400 MHz, DMSO-d6, room temperature) of Nap-NH2. Fig. S11. 13C NMR spectrum (100 MHz, DMSO-d6, room temperature) of Nap-NH2. Fig. S12. ESI IT-TOF results of Nap-NH2. [M + H]+ = 218.1142. Fig. S13. 1H NMR spectrum (400 MHz, DMSO-d6, room temperature) of Nap-DFO. Fig. S14. 13C NMR spectrum (100 MHz, DMSO-d6, room temperature) of Nap-DFO. Fig. S15. ESI IT-TOF result of Nap-DFO. [M – H]– = 775.3225. Fig. S16. 1H NMR spectra of a Nap-PEG, b Nap-PEG + CB[8] and c CB[8] in D2O. Fig. S17. CLSM images of HepG2 cells cultured with DOX∙HCl for 4 h and 9 h, respectively. The right images are the enlarged ones at the same time point. Fig. S18. Cytotoxicity evaluation of CB[8] against HepG2 cells using an MTT assay. Fig. S19. Cytotoxicity evaluation of PCL-MV against HepG2 cells using an MTT assay. Fig. S20. Cytotoxicity evaluation of Nap-PEG against HepG2 cells using an MTT assay. Fig. S21. Cytotoxicity evaluation of DOX·HCl and SNM@DOX against HepG2 cells after 24 h incubation using a CCK-8 assay. Fig. S22. Release profiles of Nap-DFO from the nanoformulation in water or PBS. Fig. S23. Time-dependent biodistribution of 89Zr SNM@DOX in liver and tumor. Fig. S24. Body weight changes of the mice treated with different formulations. Fig. S25. H&E staining of the heart tissues from a healthy mouse and b the mouse treated with SNM@DOX at day 18. [file 12951_2021_1076_MOESM1_ESM.docx]

Evaluation of the stability of cucurbit[8]uril-based ternary host−guest complexation in physiological environment and the fabrication of a supramolecular theranostic nanomedicine

Han Wu^1^, Zuobing Chen^2*^, Shaolong Qi^1^, Bing Bai^2^, Jiajun Ye^3^, Dan Wu^4^, Jie Shen^5^, Fei Kang^3*^, Guocan Yu^1*^

^1^ Key Laboratory of Organic Optoelectronics and Molecular Engineering, Department of Chemistry, Tsinghua University, Beijing 100084, P. R. China

Email: [guocanyu@mail.tsinghua.edu.cn](mailto:guocanyu@mail.tsinghua.edu.cn)

^2^ Department of Rehabilitation Medicine, the First Affiliated Hospital, College of Medicine, Zhejiang University, Hangzhou, 310003, P. R. China

E-mail: [czb1971@zju.edu.cn](mailto:czb1971@zju.edu.cn)

^3^ Department of Nuclear Medicine, Xijing Hospital, Fourth Military Medical University, Xi'an, 400030, P. R. China

Email: fmmukf@qq.com

^4^ College of Materials Science and Engineering, Zhejiang University of Technology, Hangzhou, 310014, P. R. China

^5^ Department of Pharmacy, School of Medicine, Zhejiang University City College, Hangzhou 310015, P. R. China

*
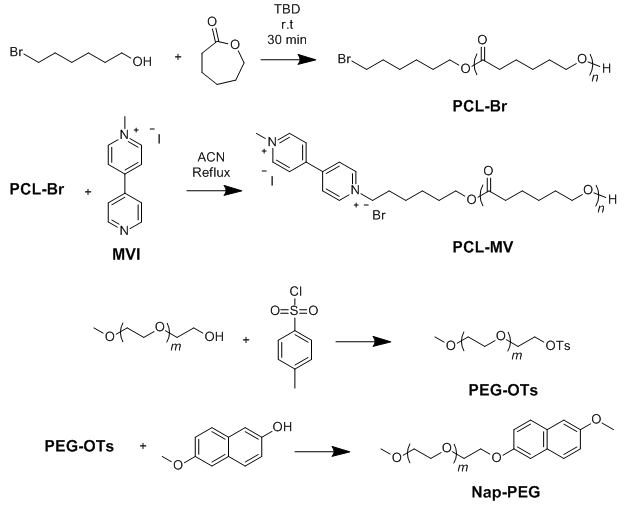
*

**Scheme S1** Synthetic routes to PCL-MV and Nap-PEG.


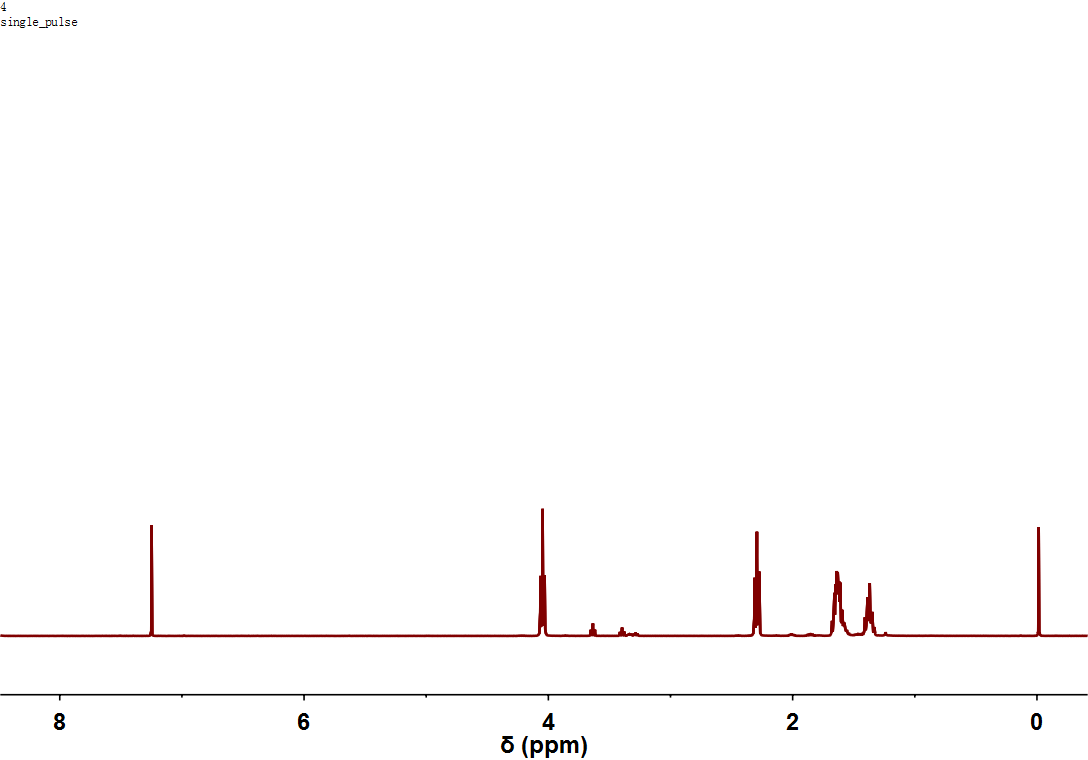


**Fig. S1** ^1^H NMR spectrum (400 MHz, CDCl_3_, room temperature) of PCL-Br.


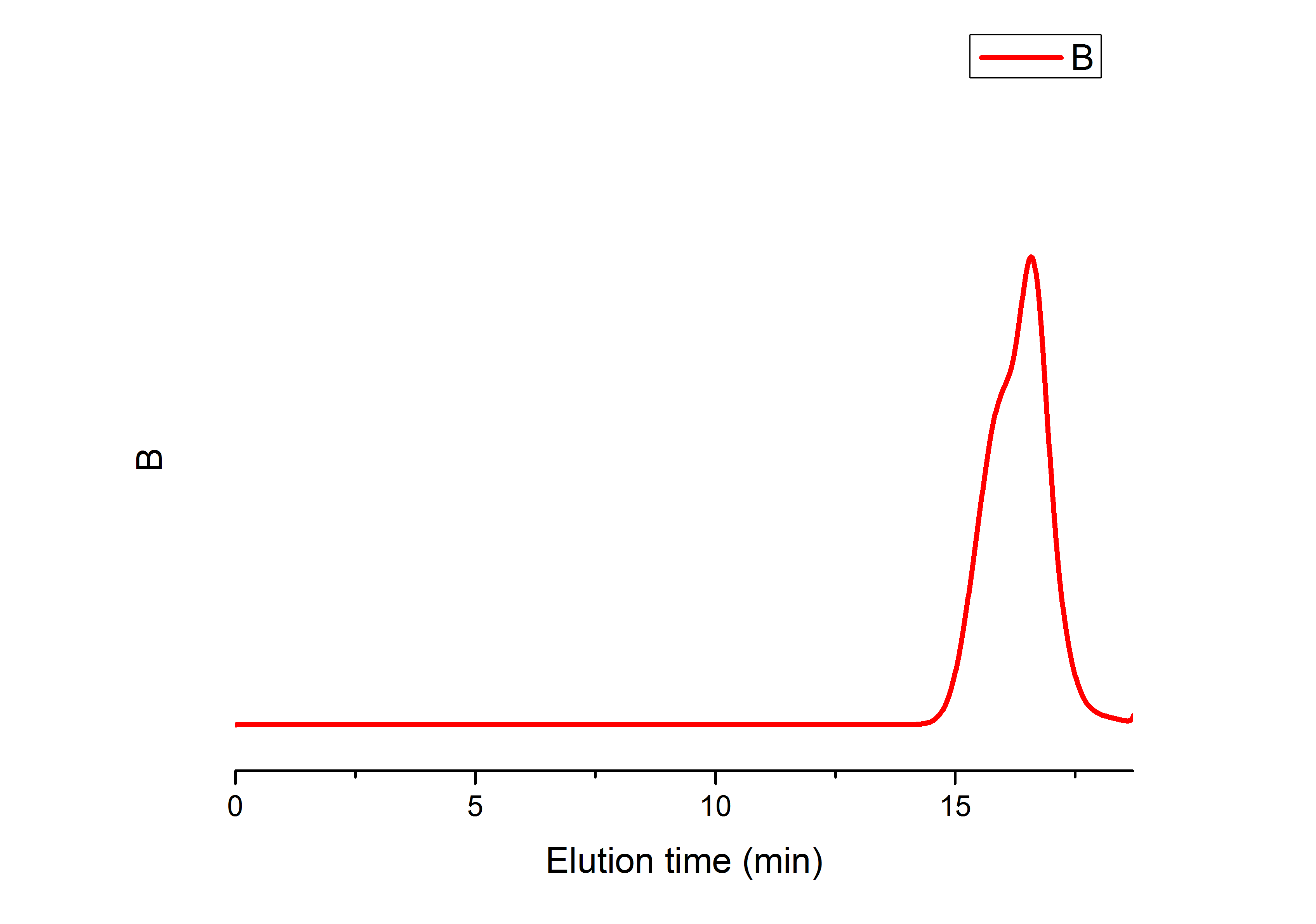


**Fig. S2** GPC curve of PCL-Br.


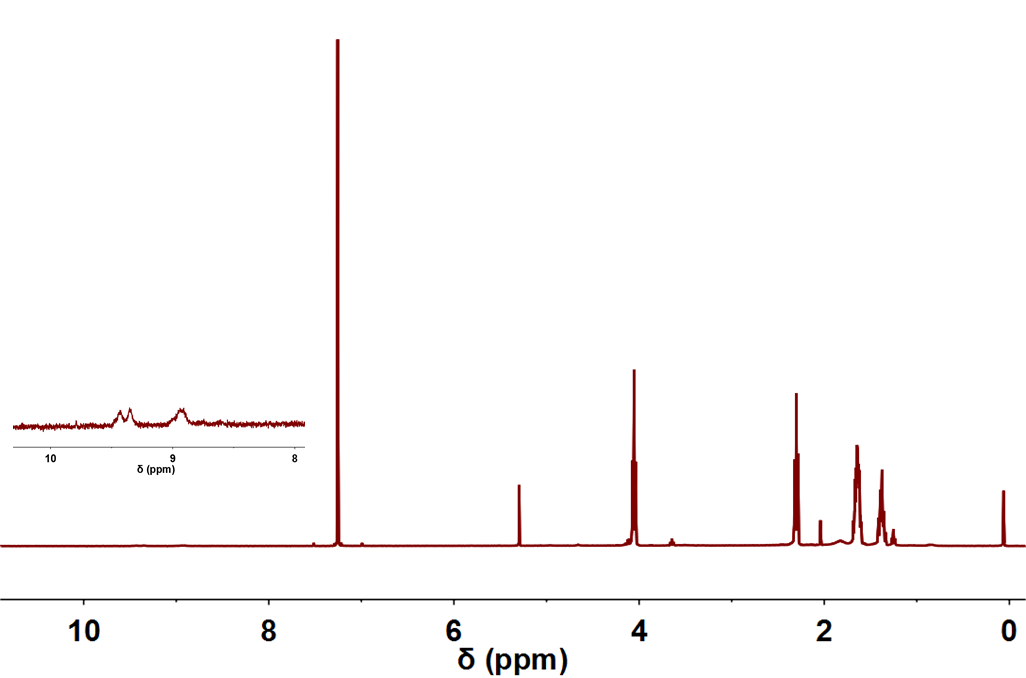


**Fig. S3** ^1^H NMR spectrum (400 MHz, CDCl_3_, room temperature) of PCL-MV.


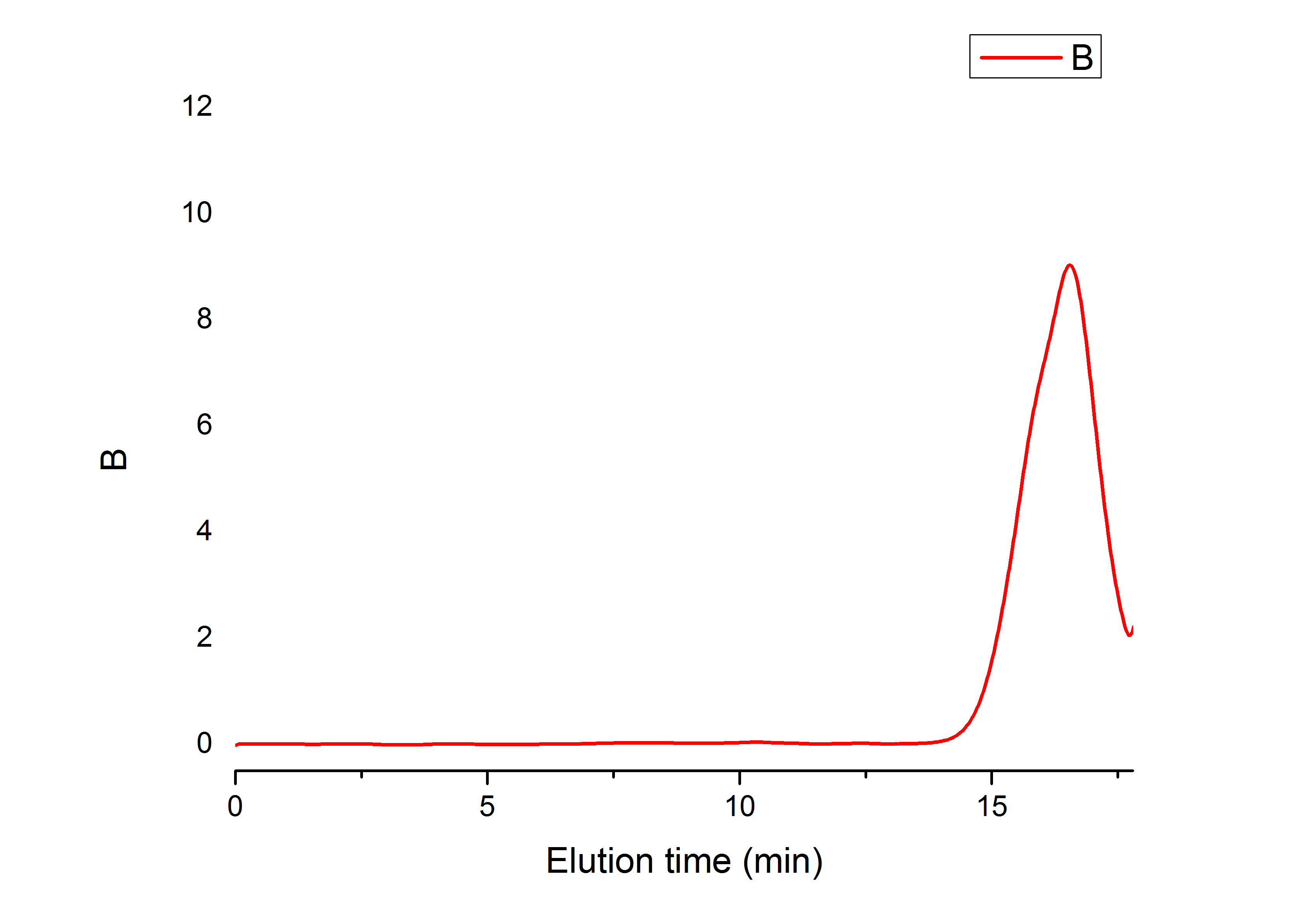


**Fig. S4** GPC curve of PCL-MV.


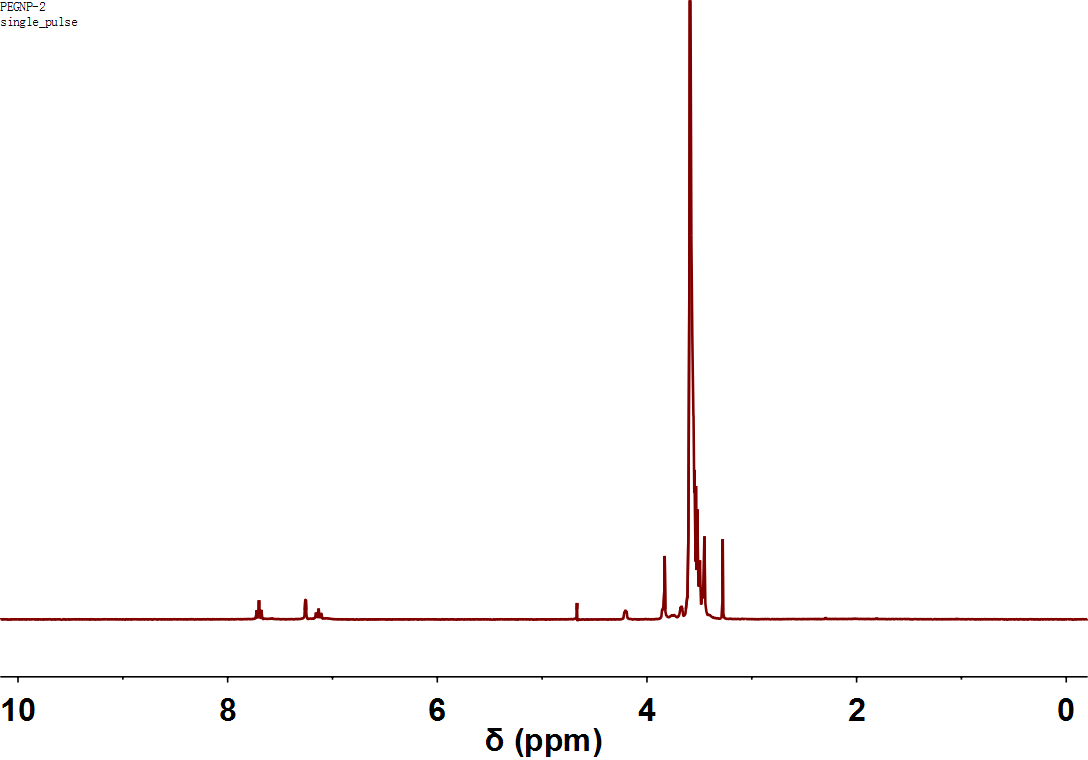


**Fig. S5** ^1^H NMR spectrum (400 MHz, CDCl_3_, room temperature) of Nap-PEG.


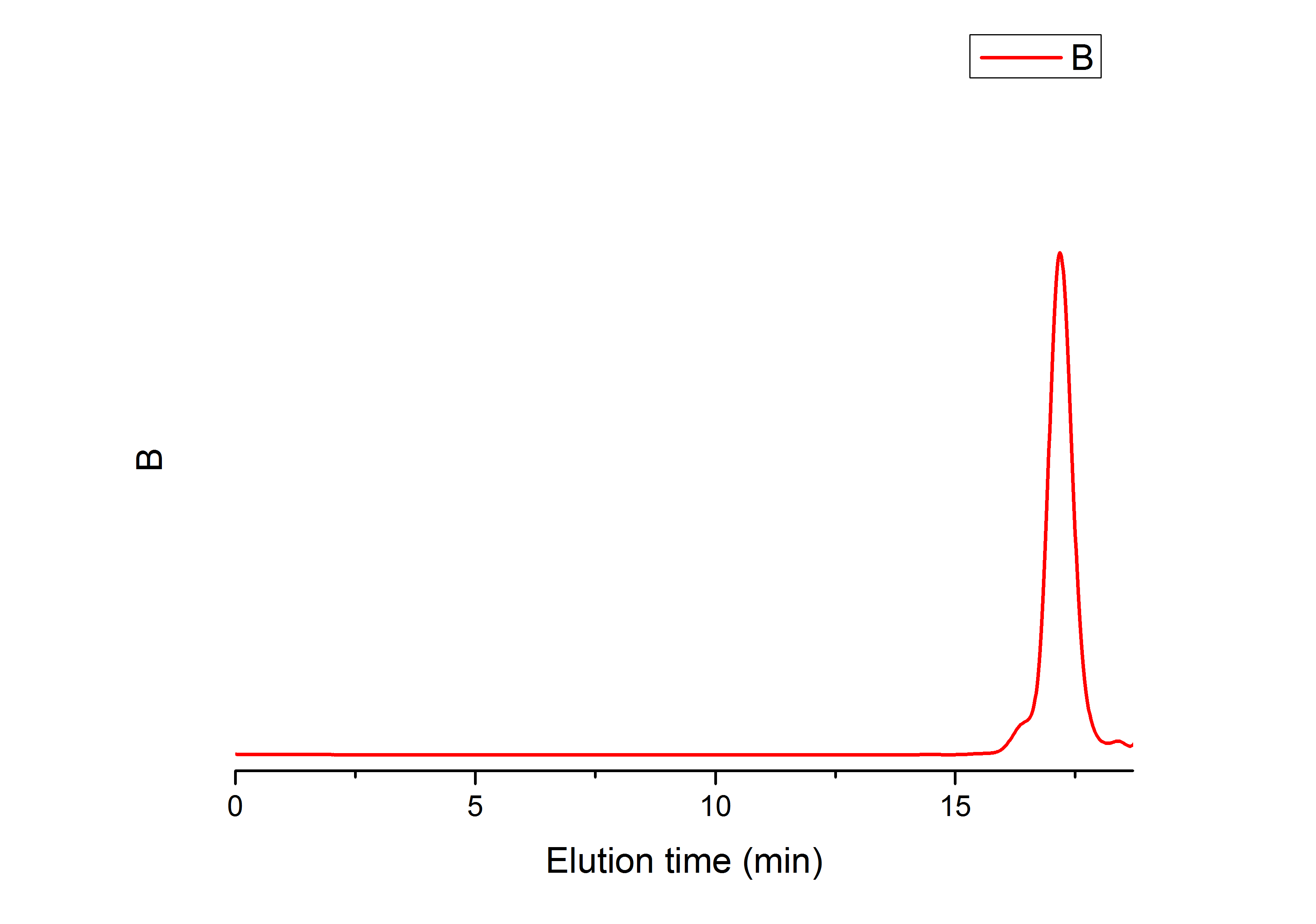


**Fig. S6** GPC curve of Nap-PEG.


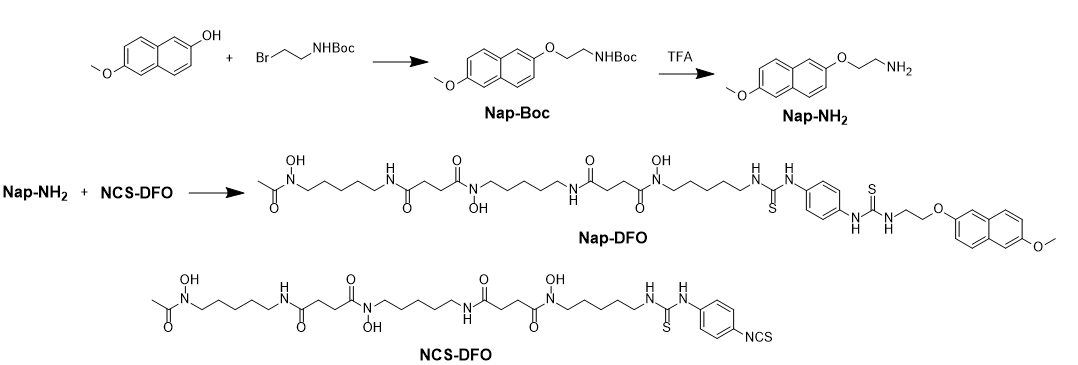


**Scheme S2** Synthetic routes to Nap-DFO.


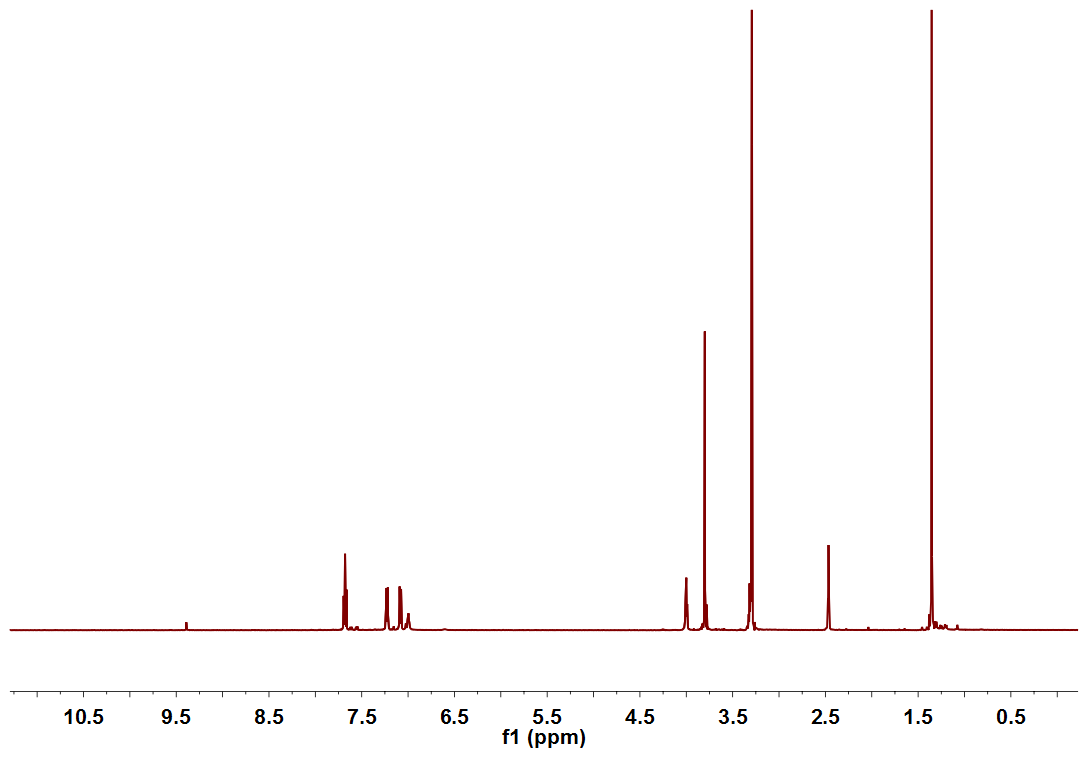


**Fig. S7** ^1^H NMR spectrum (400 MHz, CDCl_3_, room temperature) of Nap-Boc.


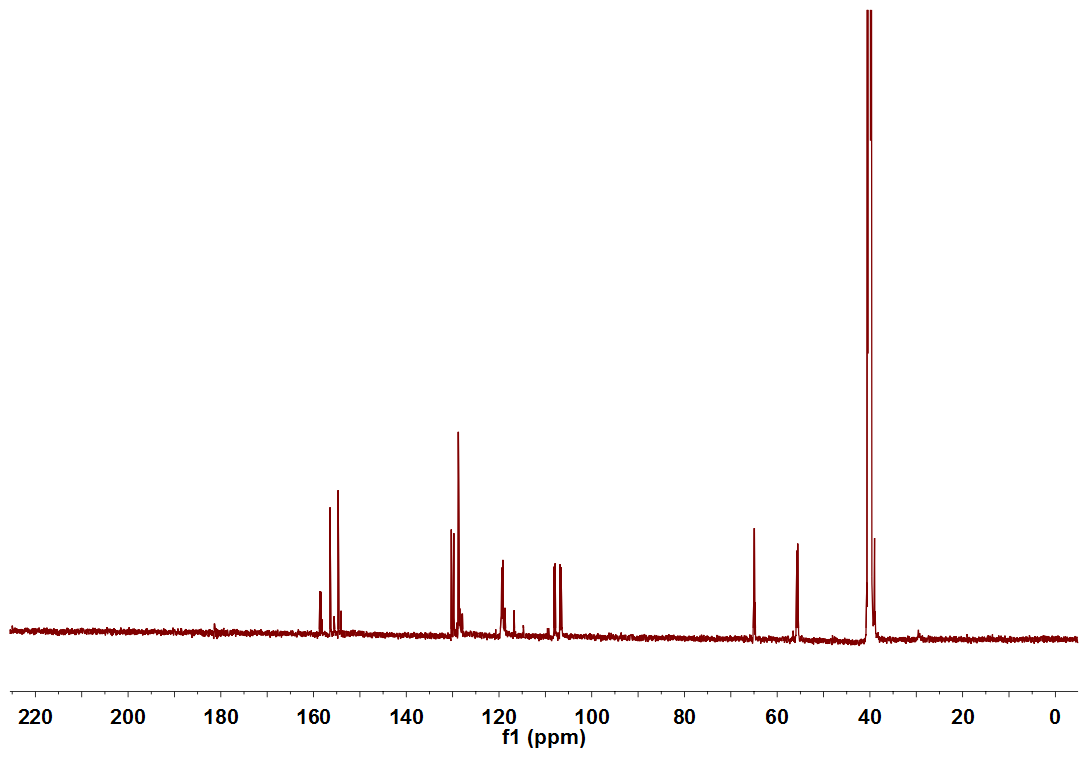


**Fig. S8** ^13^C NMR spectrum (100 MHz, CDCl_3_, room temperature) of Nap-Boc.

**Fig. S9** ESI IT-TOF results of Nap-Boc. [M + Na]^+^ = 340.1494.


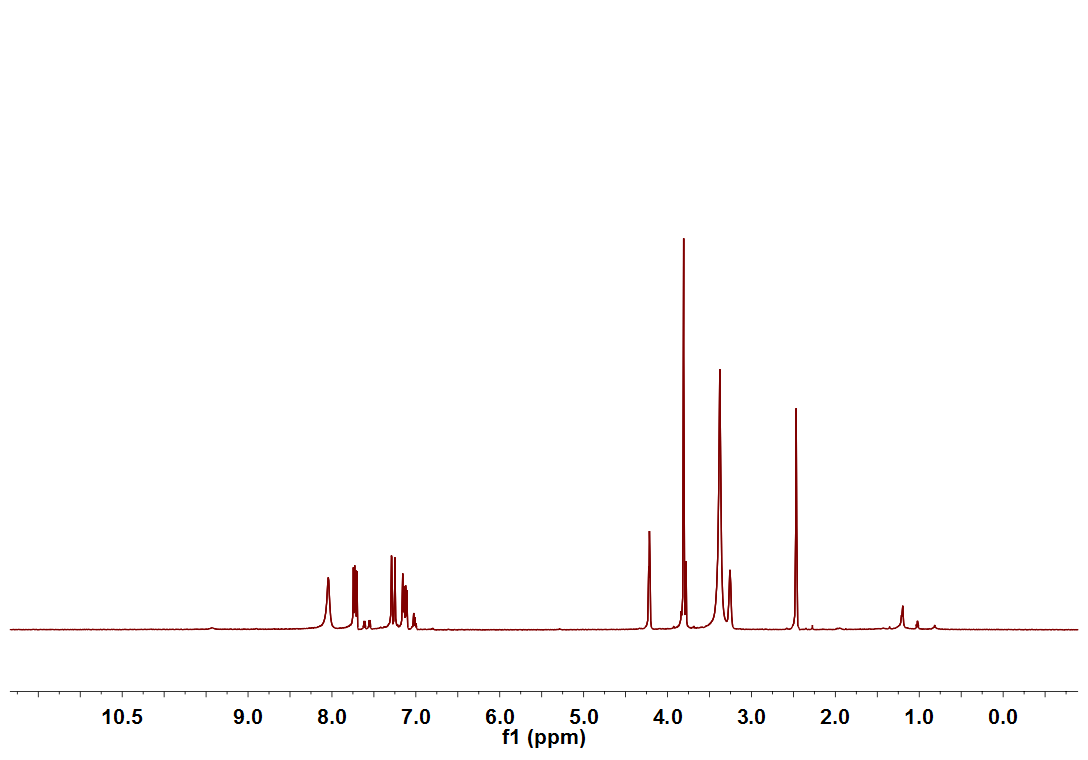


**Fig. S10** ^1^H NMR spectrum (400 MHz, DMSO-*d*_6_, room temperature) of Nap-NH_2_.


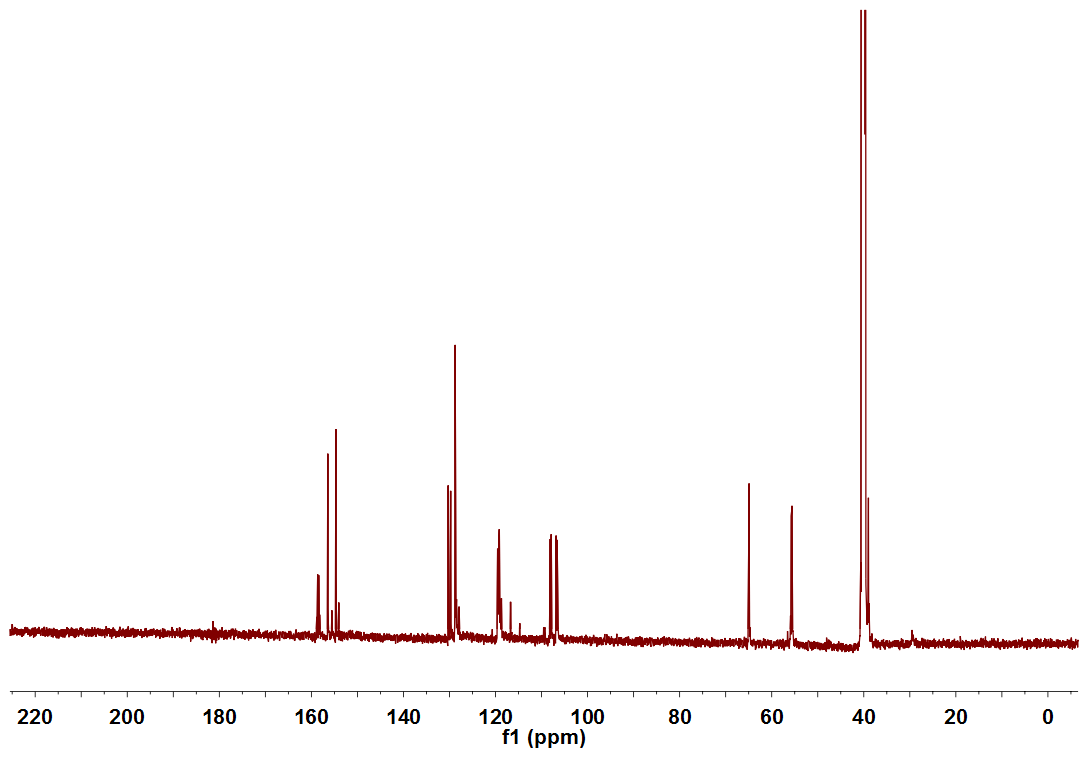


**Fig. S11** ^13^C NMR spectrum (100 MHz, DMSO-*d*_6_, room temperature) of Nap-NH_2_.

**Fig. S12** ESI IT-TOF results of Nap-NH_2_. [M + H]^+^ = 218.1142.


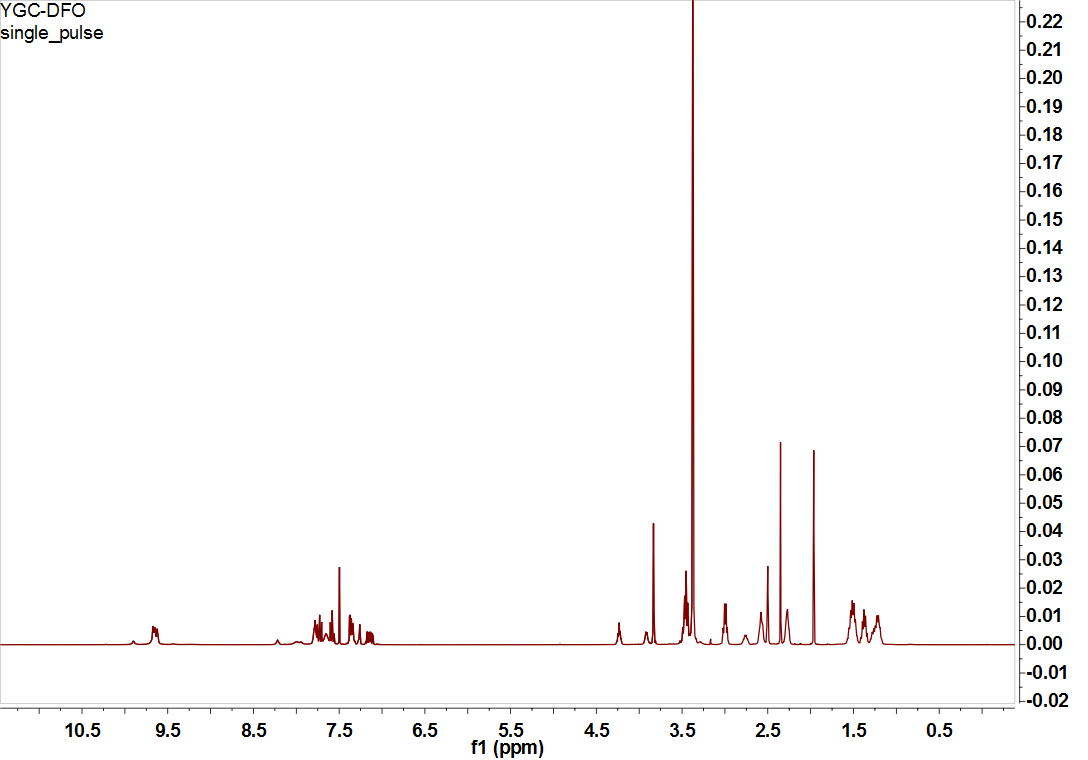


**Fig. S13** ^1^H NMR spectrum (400 MHz, DMSO-*d*_6_, room temperature) of Nap-DFO.


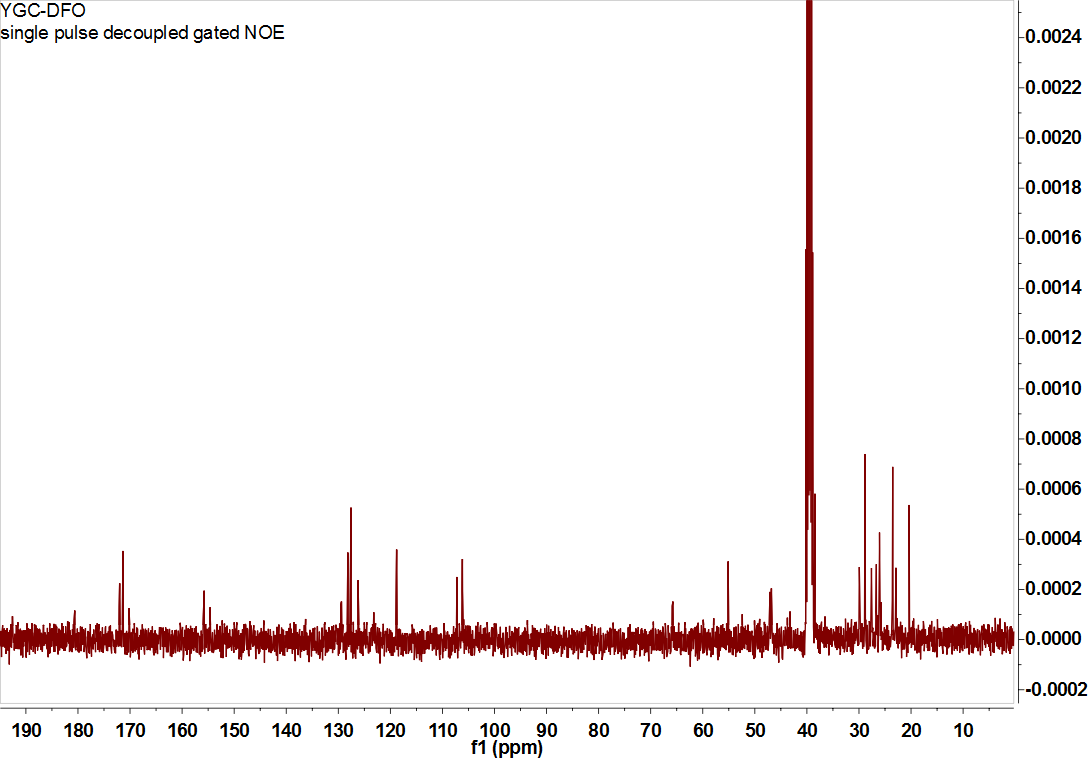


**Fig. S14** ^13^C NMR spectrum (100 MHz, DMSO-*d*_6_, room temperature) of Nap-DFO.

**Fig. S15** ESI IT-TOF result of Nap-DFO. [M – H]^–^ = 775.3225.


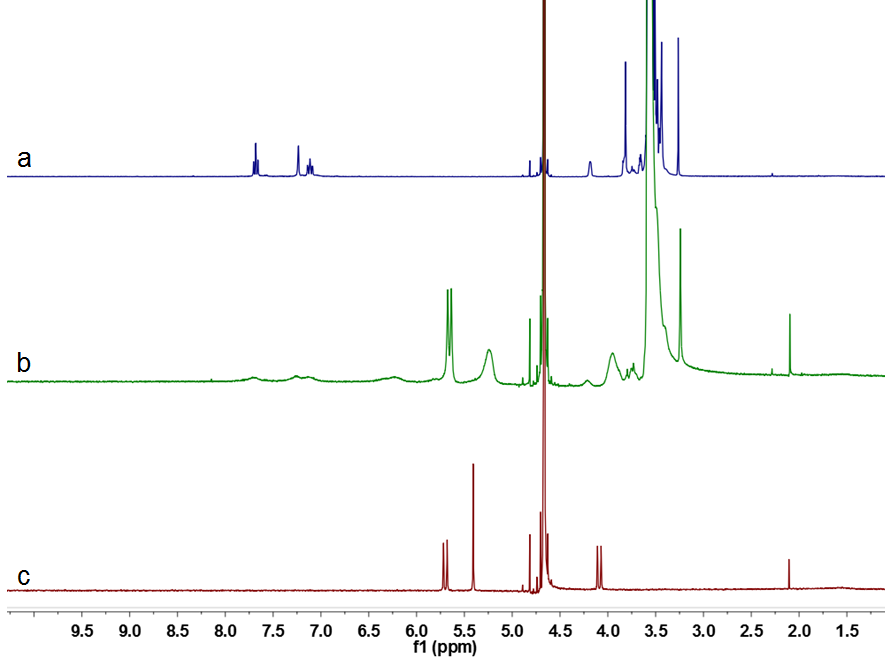


**Fig. S16** ^1^H NMR spectra of **a** Nap-PEG, **b** Nap-PEG + CB[8] and **c** CB[8] in D_2_O.


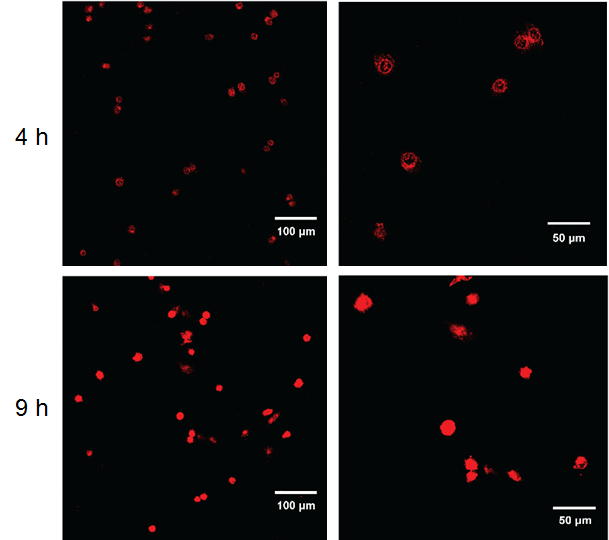


**Fig. S17** CLSM images of HepG2 cells cultured with DOX∙HCl for 4 h and 9 h, respectively. The right images are the enlarged ones at the same time point.


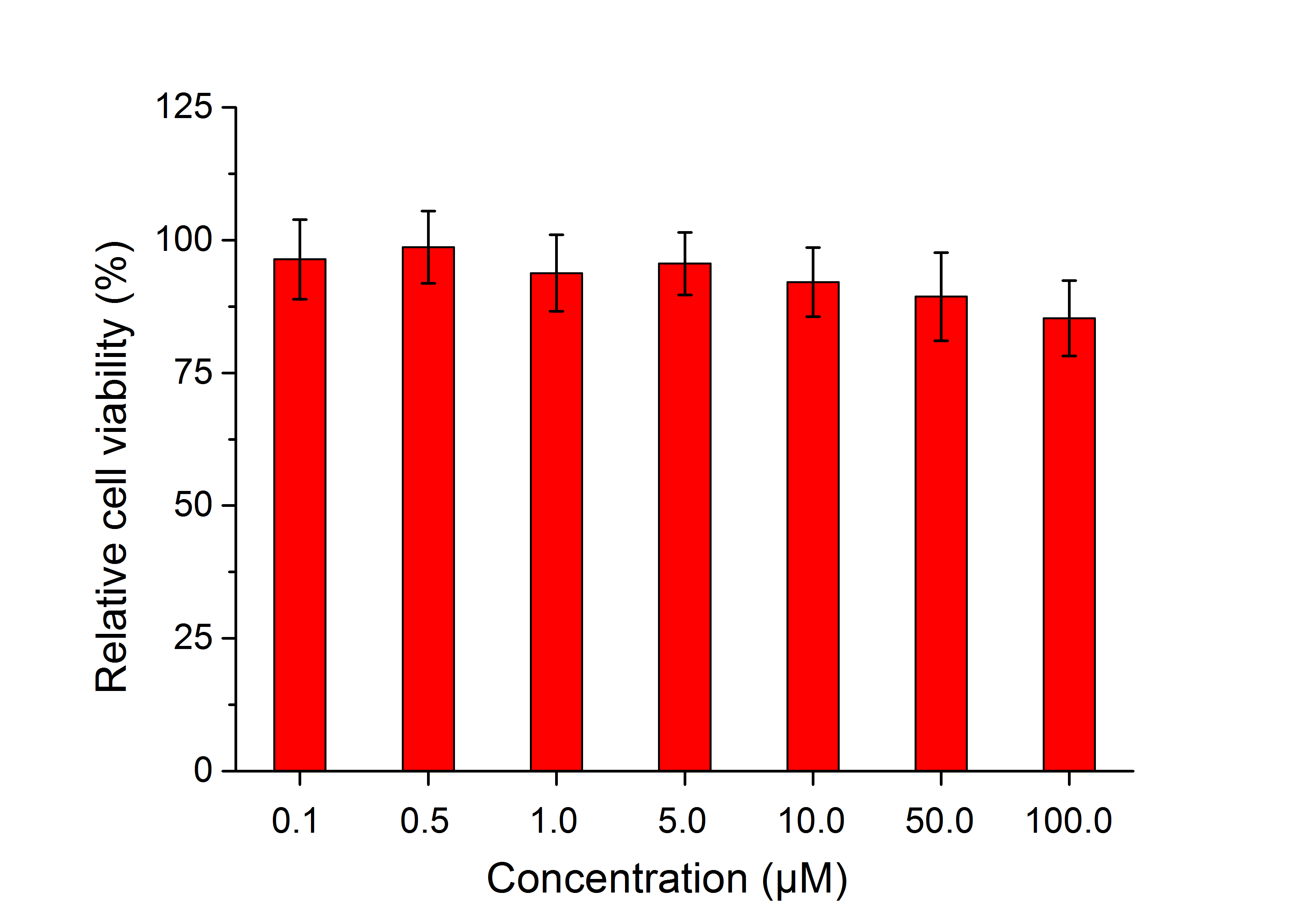


**Fig. S18** Cytotoxicity evaluation of CB[8] against HepG2 cells using an MTT assay.


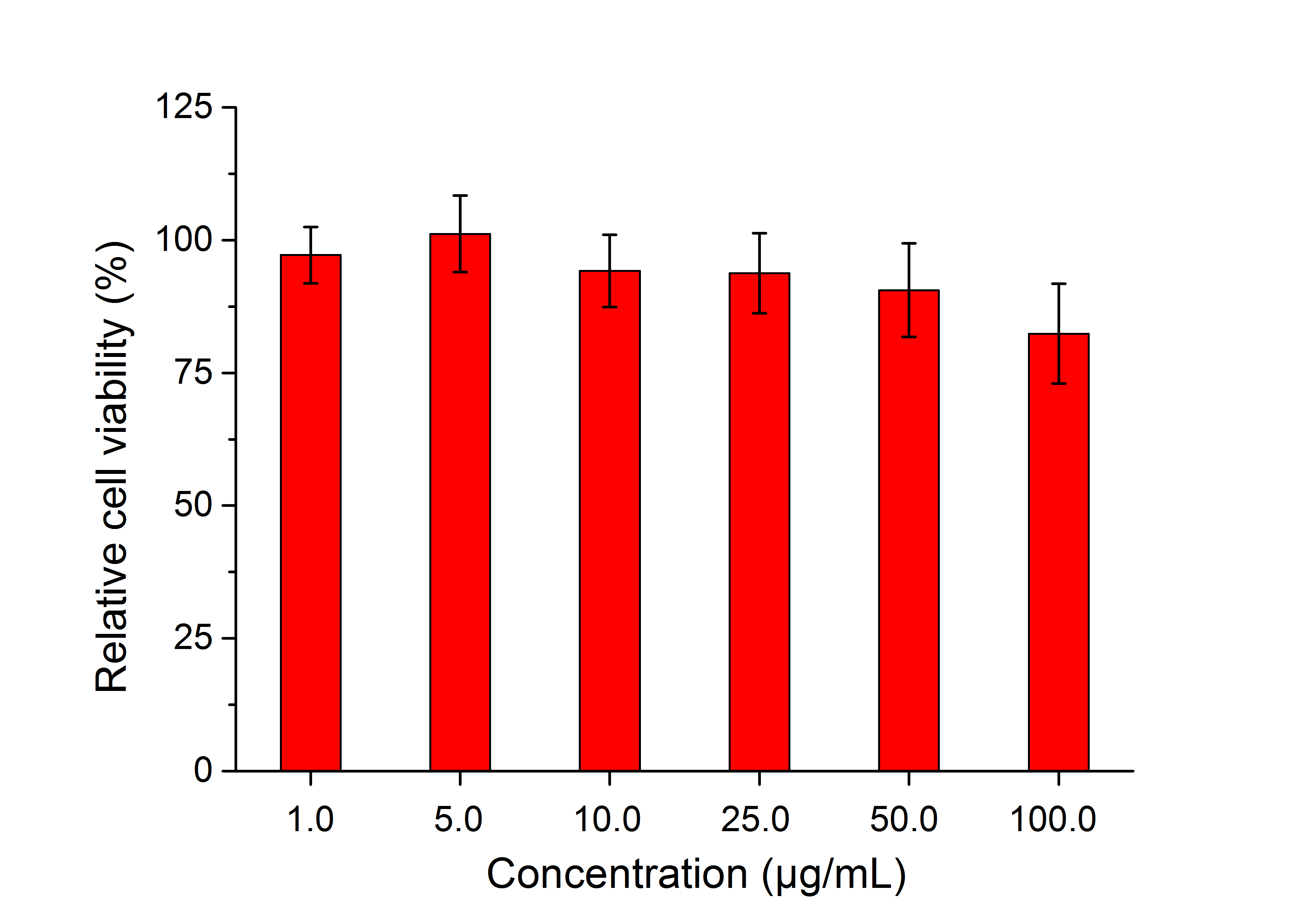


**Fig. S19** Cytotoxicity evaluation of PCL-MV against HepG2 cells using an MTT assay.


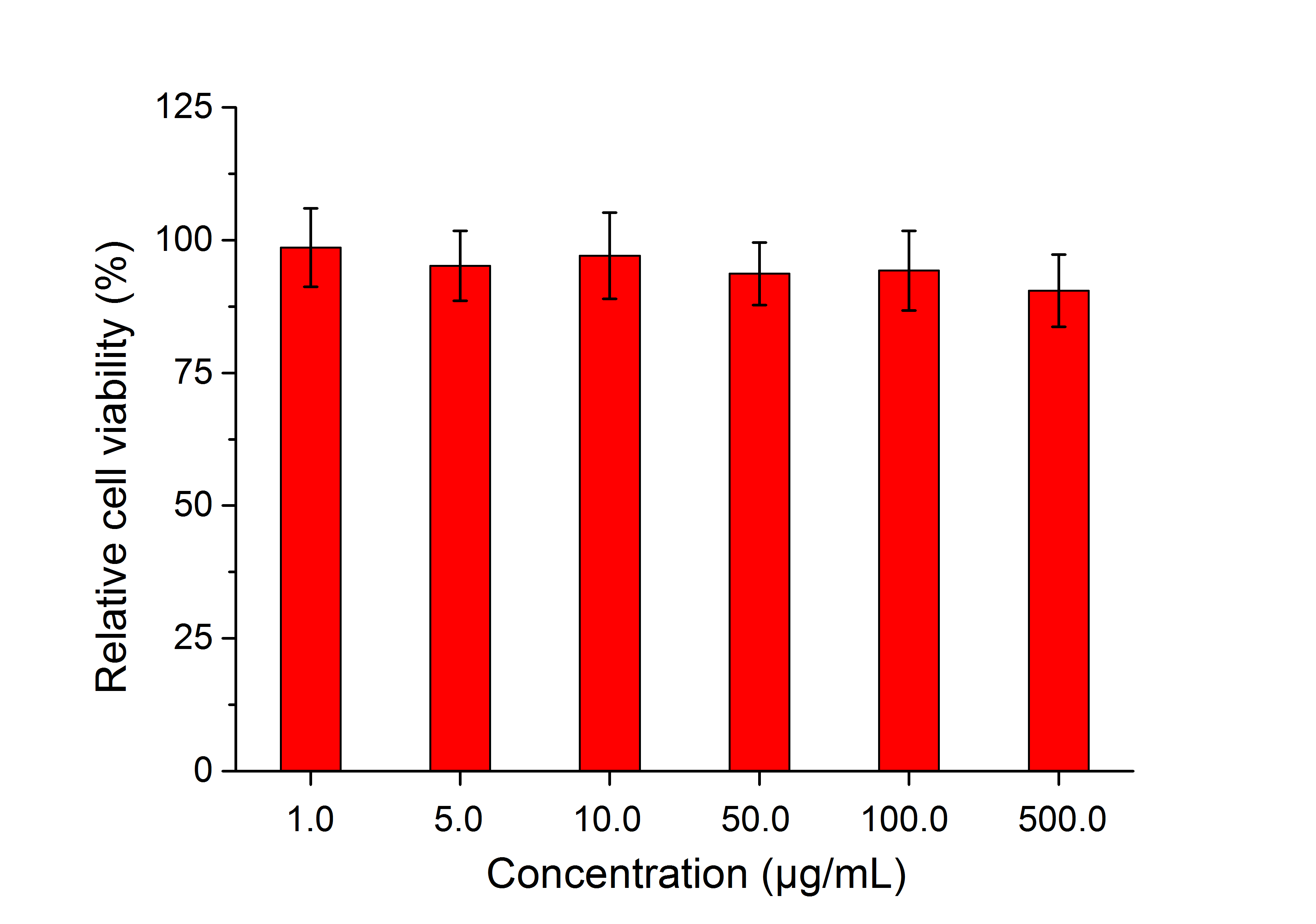


**Fig. S20** Cytotoxicity evaluation of Nap-PEG against HepG2 cells using an MTT assay.


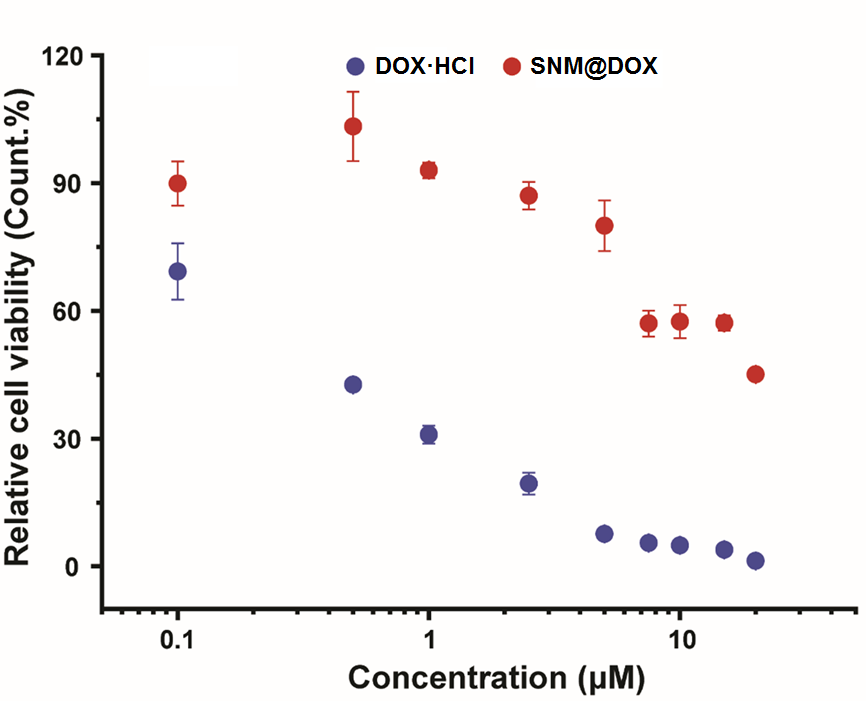


**Fig. S21** Cytotoxicity evaluation of DOX·HCl and SNM@DOX against HepG2 cells after 24 h incubation using a CCK-8 assay.


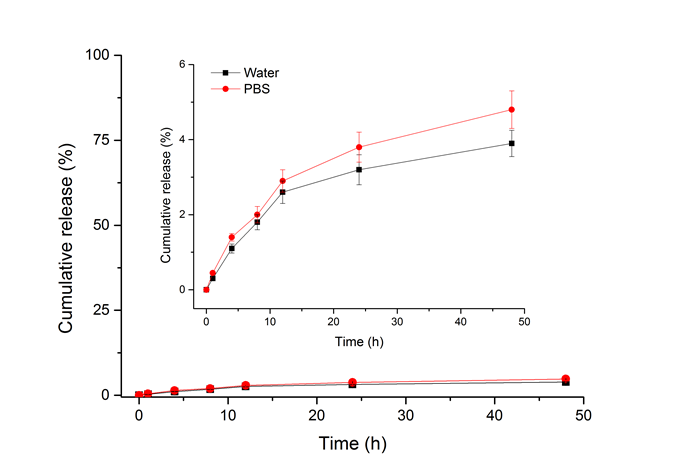


**Fig. S22** Release profiles of Nap-DFO from the nanoformulation in water or PBS.


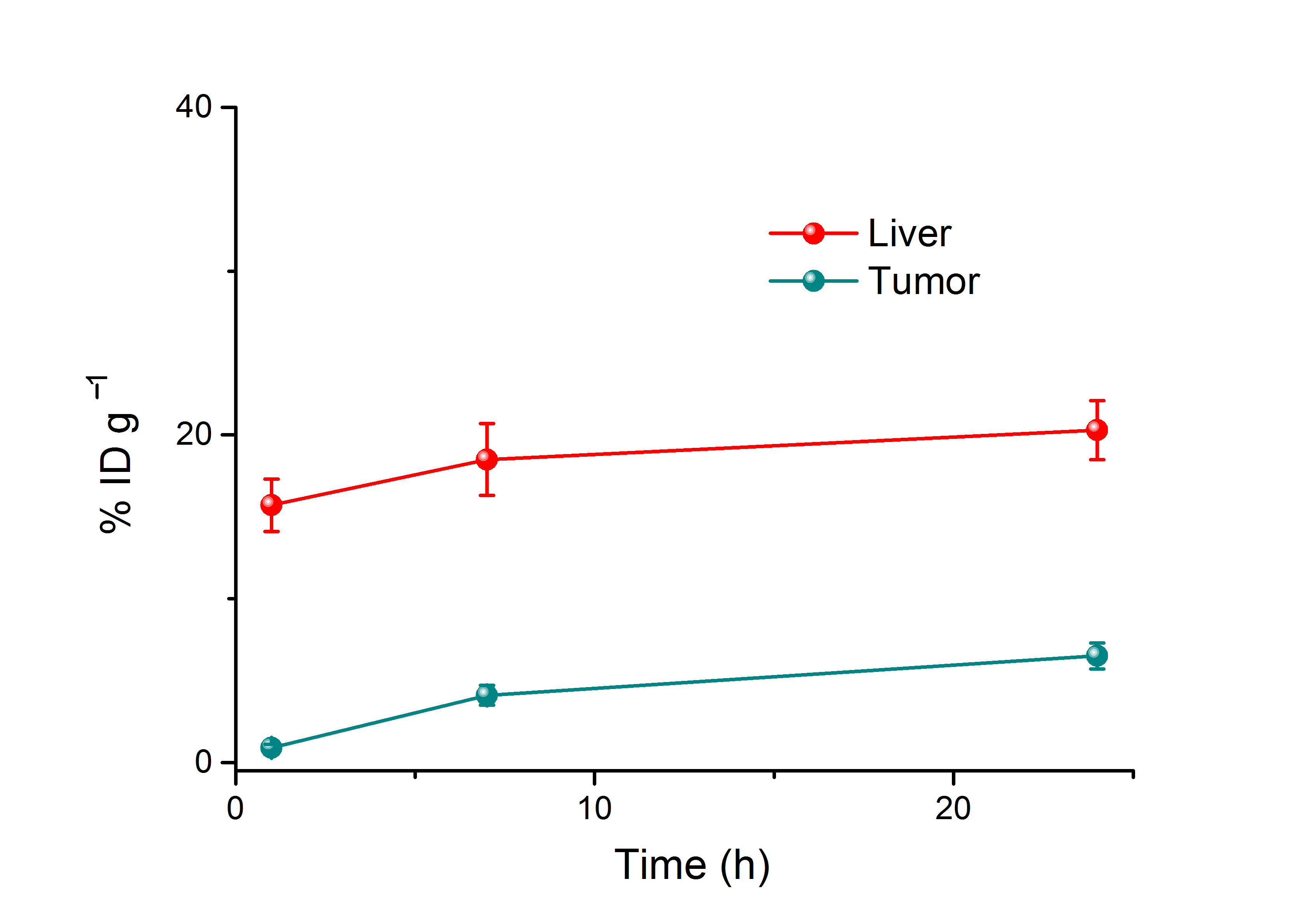


**Fig. S23** Time-dependent biodistribution of ^89^Zr SNM@DOX in liver and tumor.


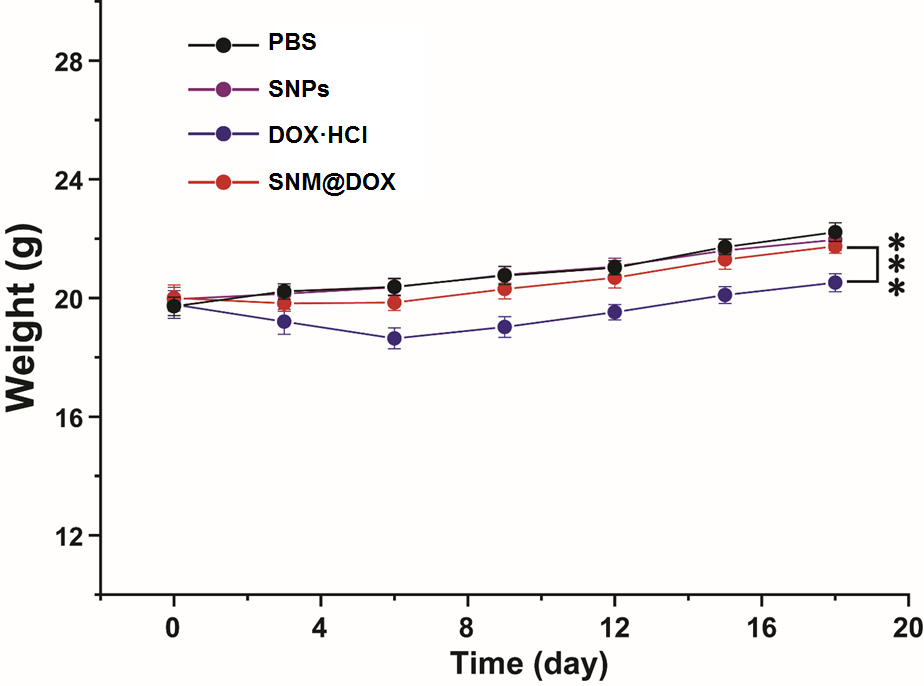


**Fig. S24** Body weight changes of the mice treated with different formulations.


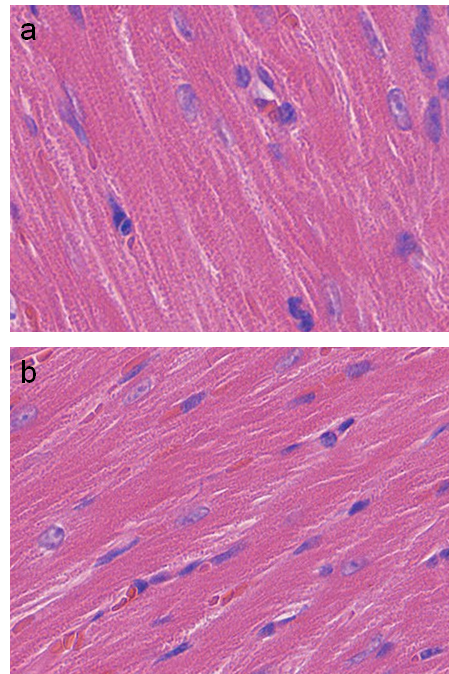


**Fig. S25** H&E staining of the heart tissues from **a** healthy mouse and **b** the mouse treated with SNM@DOX at day 18.
